# Supplementary material for: Viral coinfection promotes tuberculosis immunopathogenesis by type I IFN signaling-dependent impediment of Th1 cell pulmonary influx
Source: Nat Commun. 2022 Jun 7;13:3155. doi: 10.1038/s41467-022-30914-3 (PMC9174268; doi:10.1038/s41467-022-30914-3)
Supplement: Supplementary file 1 — Supplementary Information [file 41467_2022_30914_MOESM1_ESM.pdf]

SUPPLEMENTAL INFORMATION TITLES AND LEGENDS

Viral coinfection promotes tuberculosis immunopathogenesis by type I IFN signaling-dependent impediment of Th1 cell pulmonary influx

Tae Gun Kang[1,2#], Kee Woong Kwon[3#], Kyung Soo Kim[1,4], Insuk Lee[5], Myeong Joon Kim[1,2], Sang-Jun Ha[1,2]*, and Sung Jae Shin[3,6†]*

[1]Department of Biochemistry, College of Life Science & Biotechnology, Yonsei University, Seoul 03722, Republic of Korea

[2]Brain Korea 21 (BK21) FOUR Program, Yonsei Education & Research Center for Biosystems, Yonsei University, Seoul 03722, Republic of Korea

[3]Department of Microbiology, Graduate School of Medical Science, Brain Korea 21 Project, Yonsei University College of Medicine, Seoul 03722, Republic of Korea

[4]Institute for Breast Cancer Precision Medicine, Yonsei University College of Medicine, Seoul, Republic of Korea

[5]Department of Biotechnology, College of Life Science & Biotechnology, Yonsei University, Seoul 03722, Republic of Korea

[6]Institute for Immunology and Immunological Disease, Yonsei University College of Medicine, Seoul 03722, Republic of Korea

[#]These authors contributed equally

[†]Lead contact

***CORRESPONDENCE:** sjha@yonsei.ac.kr, sjshin@yuhs.ac

Sang-Jun Ha, Department of Biochemistry, College of Life Science & Biotechnology, Yonsei University, Seoul 03722, Republic of Korea. Phone: 82-2-2123-2696

Sung Jae Shin, Department of Microbiology, Yonsei University College of Medicine, Seoul 03722, Republic of Korea. Phone: 82-2-2228-1813

This file contains:

- Supplementary Figure 1
- Supplementary Figure 2
- Supplementary Figure 3
- Supplementary Figure 4
- Supplementary Figure 5
- Supplementary Figure 6
- Supplementary Figure 7
- Supplementary Figure 8
- Supplementary Figure 9
- Supplementary Figure 10
- Supplementary Figure 11
- Supplementary Figure 12
- Supplementary Figure 13
- Supplementary Figure 14

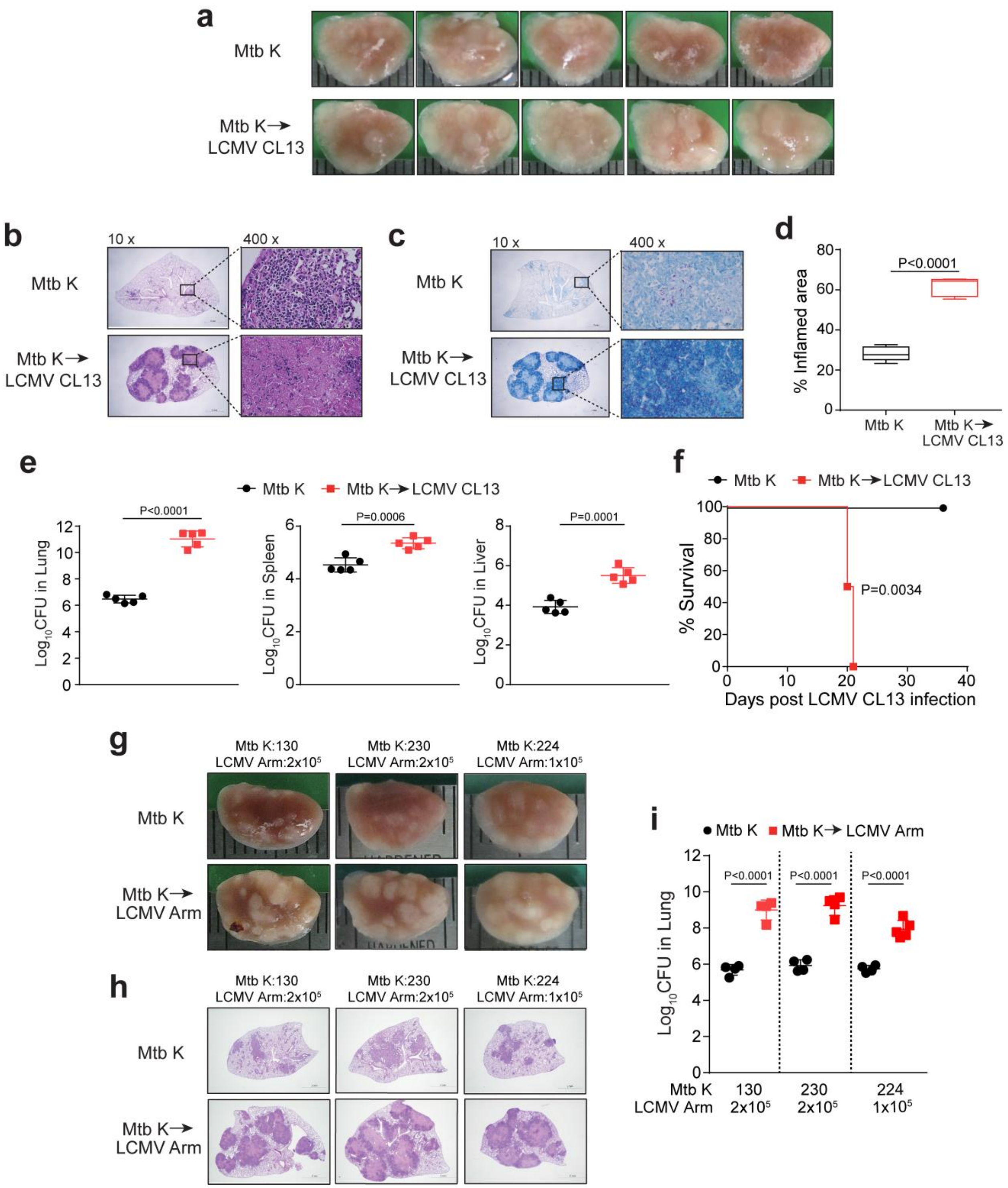

a
Mtb K
Mtb K→ LCMV CL13
b
10 x
400 x
Mtb K
Mtb K→ LCMV CL13
c
10 x
400 x
Mtb K
Mtb K→ LCMV CL13
d
% Inflamed area
P<0.0001
Mtb K
Mtb K→ LCMV CL13
e
Mtb K
Mtb K→LCMV CL13
Log10CFU in Lung
P<0.0001
Log10CFU in Spleen
P=0.0006
Log10CFU in Liver
P=0.0001
f
Mtb K
Mtb K→LCMV CL13
% Survival
P=0.0034
Days post LCMV CL13 infection
g
Mtb K:130 LCMV Arm:2x10^5
Mtb K:230 LCMV Arm:2x10^5
Mtb K:224 LCMV Arm:1x10^5
Mtb K
Mtb K→ LCMV Arm
h
Mtb K:130 LCMV Arm:2x10^5
Mtb K:230 LCMV Arm:2x10^5
Mtb K:224 LCMV Arm:1x10^5
Mtb K
Mtb K→ LCMV Arm
i
Mtb K
Mtb K→ LCMV Arm
Log10CFU in Lung
P<0.0001
P<0.0001
P<0.0001
Mtb K 130 230 224
LCMV Arm 2x10^5 2x10^5 1x10^5

Supplementary Figure 1. Necrotic lung granulomas in Mtb-infected mice subsequently challenged with LCMV CL13 or low doses of Mtb and LCMV Arm. C57BL/6 mice were infected with Mtb. Some mice from each group were subsequently infected 14 days later with LCMV CL13. At 31 days post Mtb infection, the mice were sacrificed. **a,** Gross pathology of the lung. **b,** H&E staining or **c,** acid-fast staining of the lung in each group (10x, scale bars, 2 mm; 400x, scale bars, 50 μm). **d,** The data represent the percentage of the superior lobe of the right lung showing inflammation and are shown as center, bounds of box-and-whisker and percentile plots showing the minimum and maximum values (n=5). **e,** Bacterial load in the organs of each group (n=5). **f,** Survival was monitored at the indicated time points after CL13 infection (n=5). Mice were infected with indicated dose of Mtb. Some mice from each group were subsequently infected 14 days later with the indicated dose of LCMV Arm. At 31 days post Mtb infection, the mice were sacrificed. **g,** Gross pathology of the lung. **h,** H&E staining. Scale Bars, 2 mm. **i,** Bacterial load in the lung (n=4 or 5). **d, e, i,** The data were analyzed by two-tailed unpaired Student's *t* test, and **f,** survival graphs were analyzed using the Mantel–Cox log-rank test. Plots show the mean ± SEM. The data are representative of **a-f,** at least two independent experiments or **g-i,** a single experiment. Source data are provided as a Source Data file.

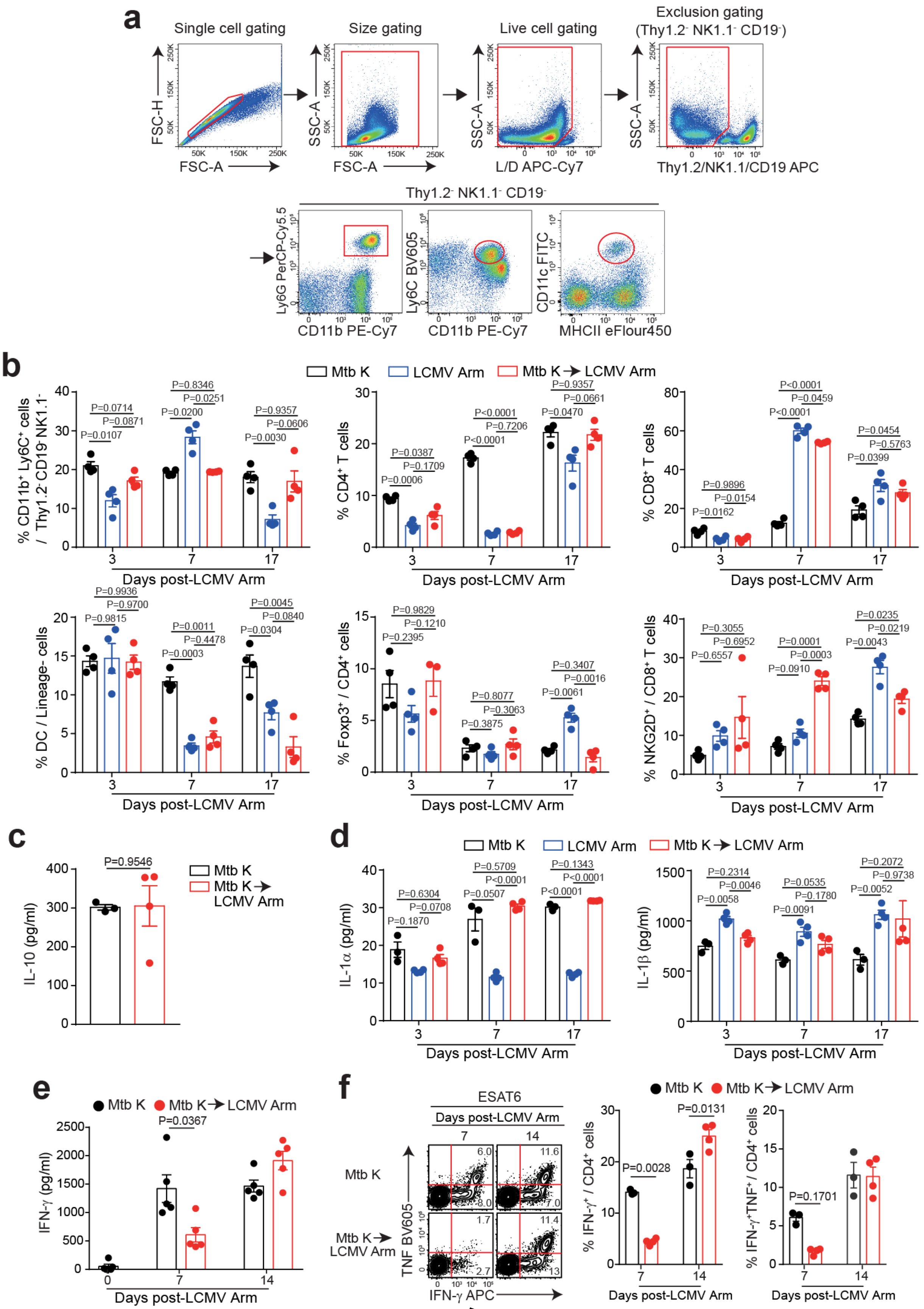
a
Single cell gating
Size gating
Live cell gating
Exclusion gating
(Thy1.2- NK1.1- CD19-)
FSC-H
FSC-A
SSC-A
L/D APC-Cy7
Thy1.2/NK1.1/CD19 APC
Thy1.2- NK1.1- CD19-
Ly6G PerCP-Cy5.5
CD11b PE-Cy7
Ly6C BV605
CD11c FITC
MHCII eFlour450
b
Mtb K
LCMV Arm
Mtb K → LCMV Arm
% CD11b+ Ly6C+ cells / Thy1.2- CD19- NK1.1-
P=0.0714
P=0.0871
P=0.0107
P=0.8346
P=0.0251
P=0.0200
P=0.9357
P=0.0606
P=0.0030
% CD4+ T cells
P=0.0387
P=0.1709
P=0.0006
P<0.0001
P=0.7206
P<0.0001
P=0.9357
P=0.0661
P=0.0470
% CD8+ T cells
P=0.9896
P=0.0154
P=0.0162
P<0.0001
P=0.0459
P<0.0001
P=0.0454
P=0.5763
P=0.0399
Days post-LCMV Arm
% DC / Lineage- cells
P=0.9936
P=0.9700
P=0.9815
P=0.0011
P=0.4478
P=0.0003
P=0.0045
P=0.0840
P=0.0304
% Foxp3+ / CD4+ cells
P=0.9829
P=0.1210
P=0.2395
P=0.8077
P=0.3063
P=0.3875
P=0.3407
P=0.0016
P=0.0061
% NKG2D+ / CD8+ T cells
P=0.3055
P=0.6952
P=0.6557
P=0.0001
P=0.0003
P=0.0910
P=0.0235
P=0.0219
P=0.0043
c
IL-10 (pg/ml)
P=0.9546
Mtb K
Mtb K → LCMV Arm
d
Mtb K
LCMV Arm
Mtb K → LCMV Arm
IL-1α (pg/ml)
P=0.6304
P=0.0708
P=0.1870
P=0.5709
P<0.0001
P=0.0507
P=0.1343
P<0.0001
P<0.0001
IL-1β (pg/ml)
P=0.2314
P=0.0046
P=0.0058
P=0.0535
P=0.1780
P=0.0091
P=0.2072
P=0.9738
P=0.0052
Days post-LCMV Arm
e
Mtb K
Mtb K → LCMV Arm
IFN-γ (pg/ml)
P=0.0367
Days post-LCMV Arm
f
ESAT6
Days post-LCMV Arm
Mtb K
Mtb K → LCMV Arm
TNF BV605
IFN-γ APC
6.0
3.0
11.6
7.0
1.7
2.7
11.4
13
Mtb K
Mtb K → LCMV Arm
% IFN-γ+ / CD4+ cells
P=0.0028
P=0.0131
% IFN-γ+TNF+ / CD4+ cells
P=0.1701
Days post-LCMV Arm

Supplementary Figure 2. Flow cytometry gating strategy for elucidation of myeloid cell populations in the lung and analysis of pulmonary immune cells and cytokines. a, Lungs were harvested, and cells were stained for cell surface expression of CD11b, Ly6G, and Ly6C. To define the myeloid cell population in the lung, total lung cells were pregated on single cells, FSC-A^{hi} SSC-A^{hi} (leukocytes), and live cells and excluded by Thy1.2^{+}, NK1.1^{+}, and CD19^{+} signals. **b,** C57BL/6 mice were infected with Mtb. Some mice from each group were subsequently infected 14 days later with LCMV Arm. At days 3-17 post LCMV Arm infection, immune cells in the lung were isolated and analyzed by flow cytometry. Each immune cell population is summarized in the plot (n=4). **c,** At 17 days post LCMV Arm infection, the IL-10 level in the lung was measured by ELISA (n=3 or 4). **d,** At days 3-17 post LCMV Arm infection, the IL-1α and IL-1β levels in lung homogenates were analyzed by ELISA (n=3 or 4). **e,** At the indicated time points after LCMV Arm infection, the IFN-γ level in lung homogenates was analyzed by ELISA (n=5). **f,** Lung lymphocytes were isolated at the indicated time points after LCMV Arm infection, and the frequency of IFN-γ- and TNF-producing CD4^{+} T cells was analyzed by flow cytometry (n=3 or 4). **b, d, e, f,** The data were analyzed by two-way ANOVA with *post hoc* Tukey's test or **c,** two-tailed unpaired Student's *t* test. Plots show the mean ± SEM. **b,** The data are representative of at least two experiments or **c, d, e, f,** a single experiment. Source data are provided as a Source Data file.

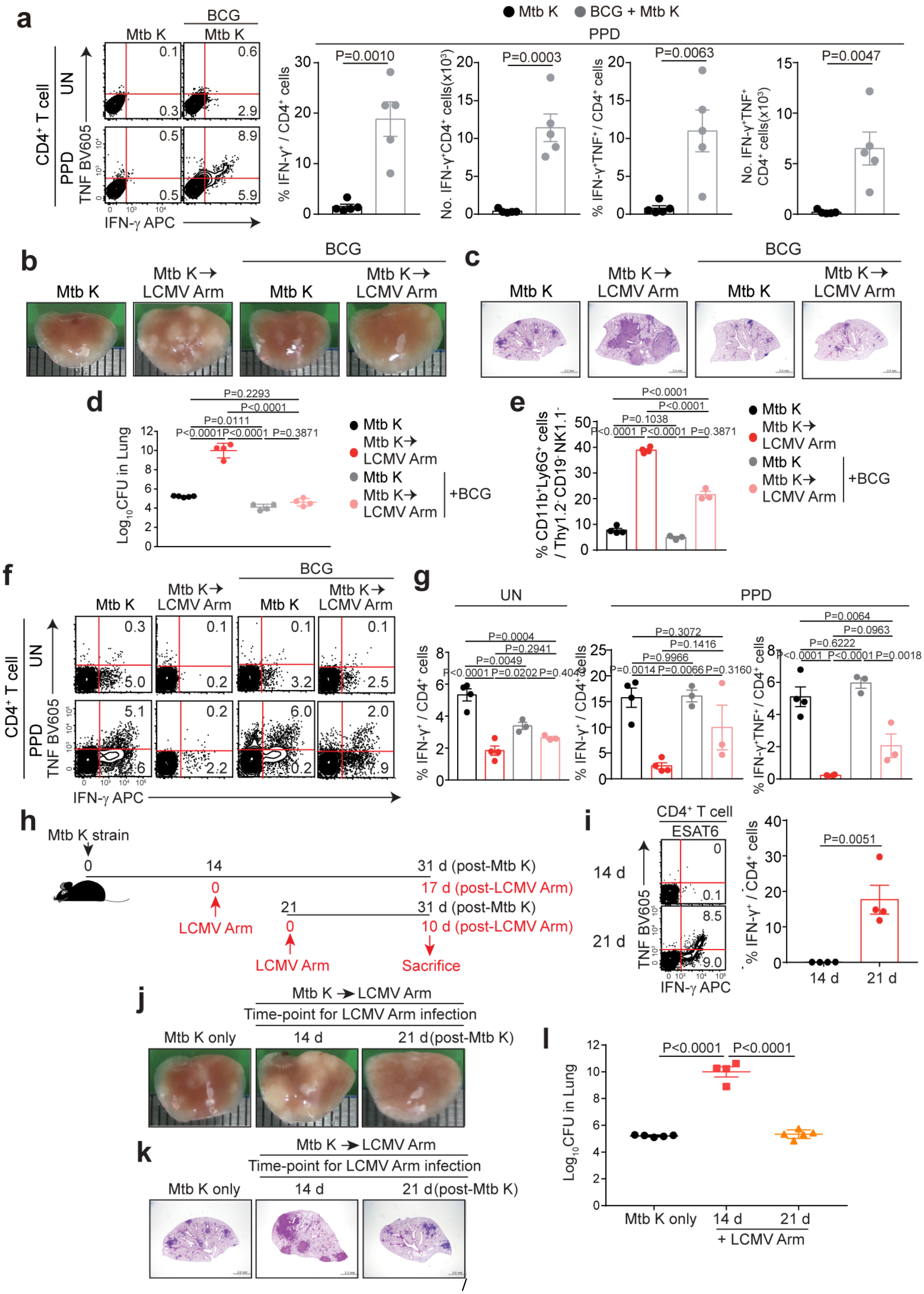
a
Mtb K
BCG + Mtb K
BCG
Mtb K
PPD
CD4+ T cell
UN
PPD
TNF BV605
IFN-γ APC
0.1
0.3
0.6
2.9
0.5
0.5
8.9
5.9
% IFN-γ+ / CD4+ cells
P=0.0010
No. IFN-γ+CD4+ cells(x10³)
P=0.0003
% IFN-γ+TNF+ / CD4+ cells
P=0.0063
No. IFN-γ+TNF+ CD4+ cells(x10³)
P=0.0047
b
BCG
Mtb K
Mtb K→ LCMV Arm
Mtb K
Mtb K→ LCMV Arm
c
BCG
Mtb K
Mtb K→ LCMV Arm
Mtb K
Mtb K→ LCMV Arm
d
Log10CFU in Lung
P=0.2293
P<0.0001
P=0.0111
P<0.0001
P<0.0001
P=0.3871
Mtb K
Mtb K→ LCMV Arm
Mtb K
Mtb K→ LCMV Arm
+BCG
e
% CD11b+Ly6G+ cells / Thy1.2-CD19-NK1.1-
P<0.0001
P<0.0001
P=0.1038
P<0.0001
P<0.0001
P=0.3871
Mtb K
Mtb K→ LCMV Arm
Mtb K
Mtb K→ LCMV Arm
+BCG
f
BCG
Mtb K
Mtb K→ LCMV Arm
Mtb K
Mtb K→ LCMV Arm
CD4+ T cell
UN
PPD
TNF BV605
IFN-γ APC
0.3
5.0
0.1
0.2
0.1
3.2
0.1
2.5
5.1
0.2
2.2
6.0
0.2
2.0
g
UN
PPD
% IFN-γ+ / CD4+ cells
P=0.0004
P=0.2941
P=0.0049
P<0.0001
P=0.0202
P=0.4043
P=0.3072
P=0.1416
P=0.9966
P=0.0014
P=0.0066
P=0.3160
% IFN-γ+TNF+ / CD4+ cells
P=0.0064
P=0.0963
P=0.6222
P<0.0001
P<0.0001
P=0.0018
h
Mtb K strain
0
14
31 d (post-Mtb K)
0
17 d (post-LCMV Arm)
LCMV Arm
21
31 d (post-Mtb K)
0
10 d (post-LCMV Arm)
LCMV Arm
Sacrifice
i
CD4+ T cell
ESAT6
14 d
21 d
0
0.1
8.5
9.0
TNF BV605
IFN-γ APC
% IFN-γ+ / CD4+ cells
P=0.0051
14 d
21 d
j
Mtb K → LCMV Arm
Time-point for LCMV Arm infection
Mtb K only
14 d
21 d(post-Mtb K)
k
Mtb K → LCMV Arm
Time-point for LCMV Arm infection
Mtb K only
14 d
21 d(post-Mtb K)
l
Log10CFU in Lung
P<0.0001
P<0.0001
Mtb K only
14 d
21 d
+ LCMV Arm

Supplementary Figure 3. Prevention of pulmonary immunopathology exacerbation by BCG vaccination or the preexistence of Mtb-specific IFN-γ-producing cells *in vivo*. C57BL/6 mice were vaccinated 3 months before challenge with Mtb. **a,** Frequencies of IFN-γ- and TNF-producing $CD4^+$ T cells in the lung were analyzed by flow cytometry at 14 days post Mtb infection (before LCMV Arm infection). **b-g,** Some mice from each group were subsequently infected with LCMV Arm at 14 days post Mtb infection. Then, mice were sacrificed at 31 days post Mtb infection. **b,** Gross pathology of the lung and **c,** H&E staining of the lung in each group. Scale bars, 2 mm. **d,** Bacterial loads in the lung (n=4 or 5). **e,** The frequency of $CD11b^+Ly6G^+$ cells at 7 days post LCMV Arm infection is summarized in the plot (n=3 or 4). **f-g,** Frequencies of IFN-γ- and TNF-producing $CD4^+$ T cells were analyzed by flow cytometry (n=3 or 4). **h,** C57BL/6 mice were infected with Mtb. Some mice from each group were subsequently infected with LCMV Arm at the indicated time points after Mtb infection. **i,** At 14 days or 21 days post Mtb infection (before LCMV Arm infection), isolated lung lymphocytes were restimulated *ex vivo* with an ESAT6 pool containing all peptides. The frequencies of IFN-γ- and TNF-producing $CD4^+$ T cells were analyzed by flow cytometry (n=4). **j-l,** At 31 days post Mtb infection, the mice were sacrificed. **j,** Gross pathology or **k,** H&E staining of the lungs in each group. Scale bars, 2 mm. **l,** Bacterial load of the lung in each group (n=4 or 5). The data were analyzed by **a,** two-tailed unpaired Student's *t* test or **d, e, g, and l,** one-way ANOVA with *post hoc* Tukey's test or **i,** two-tailed unpaired Student's *t* test. Plots show the mean ± SEM. **a-l,** The data are representative of a single experiment. Source data are provided as a Source Data file.

a

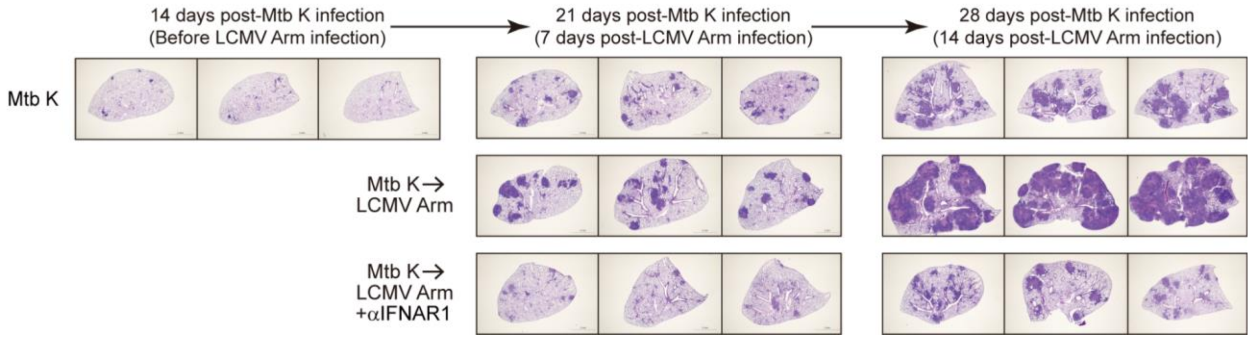
14 days post-Mtb K infection
(Before LCMV Arm infection)
21 days post-Mtb K infection
(7 days post-LCMV Arm infection)
28 days post-Mtb K infection
(14 days post-LCMV Arm infection)
Mtb K
Mtb K→
LCMV Arm
Mtb K→
LCMV Arm
+αIFNAR1

Supplementary Figure 4. Analysis of lung pathology at diverse time points. a, At -1, 1, and 3 days after LCMV Arm infection, a monoclonal anti-IFNAR-1 antibody (αIFNAR-1) was administered i.p. into coinfected mice. The mice were sacrificed at the indicated time points, and gross pathology was analyzed. The data are representative of a single experiment. Scale Bars, 2 mm

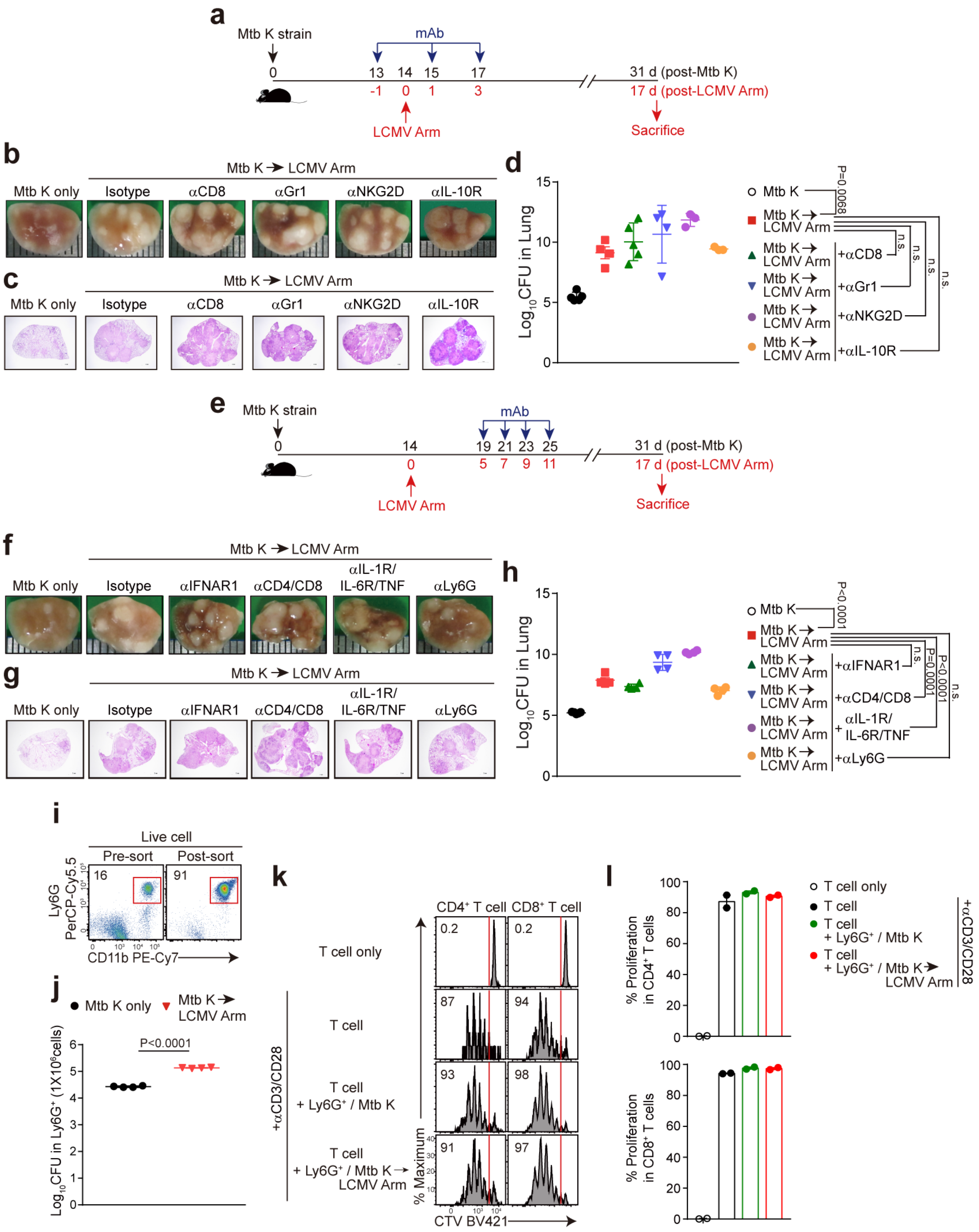
a
Mtb K strain
mAb
0
13 14 15 17
-1 0 1 3
31 d (post-Mtb K)
17 d (post-LCMV Arm)
LCMV Arm
Sacrifice
b
Mtb K ➔ LCMV Arm
Mtb K only
Isotype
αCD8
αGr1
αNKG2D
αIL-10R
c
Mtb K ➔ LCMV Arm
Mtb K only
Isotype
αCD8
αGr1
αNKG2D
αIL-10R
d
Log10CFU in Lung
Mtb K
Mtb K➔ LCMV Arm
Mtb K➔ LCMV Arm +αCD8
Mtb K➔ LCMV Arm +αGr1
Mtb K➔ LCMV Arm +αNKG2D
Mtb K➔ LCMV Arm +αIL-10R
P=0.0068
n.s.
e
Mtb K strain
mAb
0
14
19 21 23 25
0
5 7 9 11
31 d (post-Mtb K)
17 d (post-LCMV Arm)
LCMV Arm
Sacrifice
f
Mtb K ➔ LCMV Arm
Mtb K only
Isotype
αIFNAR1
αCD4/CD8
αIL-1R/ IL-6R/TNF
αLy6G
g
Mtb K ➔ LCMV Arm
Mtb K only
Isotype
αIFNAR1
αCD4/CD8
αIL-1R/ IL-6R/TNF
αLy6G
h
Log10CFU in Lung
Mtb K
Mtb K➔ LCMV Arm
Mtb K➔ LCMV Arm +αIFNAR1
Mtb K➔ LCMV Arm +αCD4/CD8
Mtb K➔ LCMV Arm +αIL-1R/ IL-6R/TNF
Mtb K➔ LCMV Arm +αLy6G
P<0.0001
n.s.
i
Live cell
Pre-sort
Post-sort
16
91
Ly6G PerCP-Cy5.5
CD11b PE-Cy7
j
Mtb K only
Mtb K➔ LCMV Arm
P<0.0001
Log10CFU in Ly6G+ (1X10^6cells)
k
CD4+ T cell
CD8+ T cell
T cell only
0.2
0.2
+αCD3/CD28
T cell
87
94
T cell + Ly6G+ / Mtb K
93
98
T cell + Ly6G+ / Mtb K➔ LCMV Arm
91
97
% Maximum
CTV BV421
l
% Proliferation in CD4+ T cells
% Proliferation in CD8+ T cells
T cell only
T cell
T cell + Ly6G+ / Mtb K
T cell + Ly6G+ / Mtb K➔ LCMV Arm
+αCD3/CD28

Supplementary Figure 5. Effect of blockade or depletion of immunological factors on pulmonary pathology and analysis of the characteristics of CD11b$^+$Ly6G$^+$ populations. a-h, C57BL/6 mice were infected with Mtb. Some mice from each group were subsequently infected 14 days later with LCMV Arm. At the indicated time points, a monoclonal antibody was administered i.p. into coinfected mice. The mice were sacrificed at 31 days post Mtb infection. **b, f,** Gross pathology or **c, g,** H&E staining of the lung in each group. Scale Bars, 2 mm. **d, h,** Bacterial loads in the lung of each group (n=3, 4, 5). **i,** Representative cell isolation gating strategy for the CD11b$^+$Ly6G$^+$ cell population. **j,** At 4 weeks post infection, equal numbers of isolated CD11b$^+$Ly6G$^+$ cells from Mtb-infected and coinfected mice were lysed with 0.05% Triton-X 100, and the lysates were plated onto 7H10 agar to enumerate the bacteria (n=4). **k, l,** Naïve splenic T cells were stimulated with anti-CD3 and anti-CD28 monoclonal antibodies in the absence or presence of CD11b$^+$Ly6G$^+$ cells isolated from the lungs of Mtb-infected or coinfected mice at 21 days post Mtb infection. **k,** Flow cytometry data showing the proliferation of CD4$^+$ or CD8$^+$ T cells assessed by a Cell Trace Violet (CTV) dilution assay. **l,** The percentages of CTV$^+$CD4$^+$ T cells and CD8$^+$ T cells are summarized in the graph (n=2 weell/group). The data were analyzed by **d, h, l,** one-way ANOVA with *post hoc* Tukey's test or **j,** two-tailed unpaired Student's *t* test. Plots show the mean ± SEM. **a-h,** The data are representative of at least two independent experiments or **i-l,** representative of a single experiment. n.s., not significant. Source data are provided as a Source Data file.

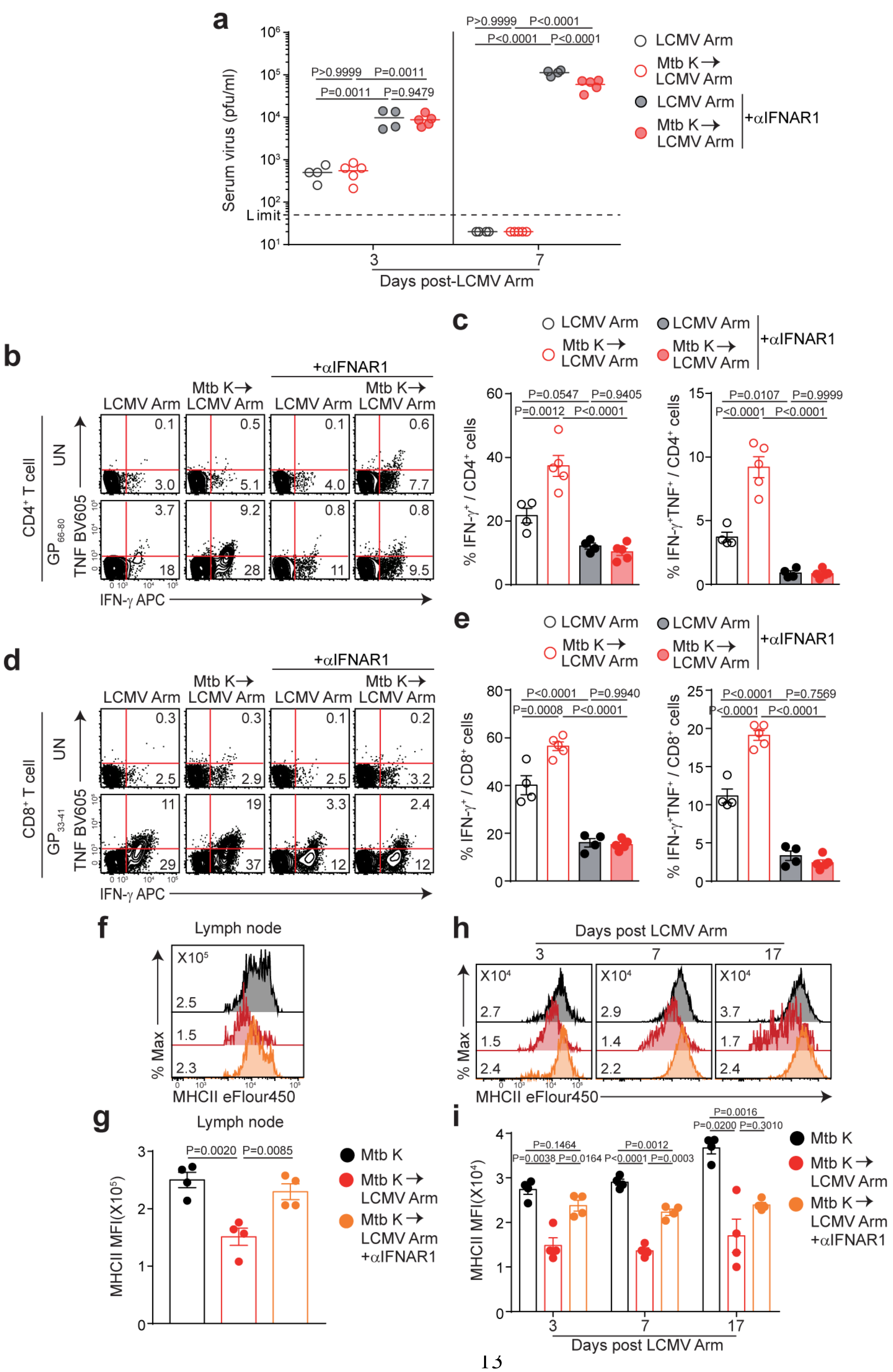

a
Serum virus (pfu/ml)
Limit
Days post-LCMV Arm
P>0.9999
P=0.0011
P=0.0011
P=0.9479
P>0.9999
P<0.0001
P<0.0001
P<0.0001
LCMV Arm
Mtb K→ LCMV Arm
LCMV Arm
Mtb K→ LCMV Arm
+αIFNAR1
b
+αIFNAR1
LCMV Arm
Mtb K→ LCMV Arm
LCMV Arm
Mtb K→ LCMV Arm
CD4+ T cell
UN
GP66-80
TNF BV605
IFN-γ APC
c
% IFN-γ+ / CD4+ cells
% IFN-γ+TNF+ / CD4+ cells
P=0.0547
P=0.9405
P=0.0012
P<0.0001
P=0.0107
P=0.9999
P<0.0001
P<0.0001
d
CD8+ T cell
GP33-41
e
% IFN-γ+ / CD8+ cells
% IFN-γ+TNF+ / CD8+ cells
P<0.0001
P=0.9940
P=0.0008
P<0.0001
P<0.0001
P=0.7569
P<0.0001
P<0.0001
f
Lymph node
% Max
MHCII eFlour450
h
Days post LCMV Arm
g
Lymph node
MHCII MFI(X10⁵)
P=0.0020
P=0.0085
Mtb K
Mtb K→ LCMV Arm
Mtb K→ LCMV Arm +αIFNAR1
i
MHCII MFI(X10⁴)
P=0.1464
P=0.0038
P=0.0164
P=0.0012
P<0.0001
P=0.0003
P=0.0016
P=0.0200
P=0.3010
Days post LCMV Arm

Supplementary Figure 6. Viral titers and LCMV-specific T cell responses during infection and the effect of type I IFN blockade on the expression level of MHCII in DCs. a, Serum was collected on the indicated days from each group of mice, and the virus titer was determined. The dashed line indicates the limit of virus detection. Samples with signals below the detection limit are represented as half of the detection limit (n=4 or 5). **b-e,** Isolated lung lymphocytes were restimulated *ex vivo* with the GP33 and GP276 peptides for $CD8^+$ T cell responses or the GP66 peptide for $CD4^+$ T cell responses. **b, d,** Frequencies of IFN-γ- and TNF-producing T cells were analyzed by flow cytometry, and **c,** the frequencies of $CD4^+$ T cells and **e,** $CD8^+$ T cells producing both IFN-γ and TNF in the lung are summarized in the plot (n=4 or 5). **f-i,** DCs were isolated from **f,** LNs at 7 days post LCMV Arm infection or **h,** lungs at the indicated days post LCMV Arm infection, and MHCII expression on DCs was analyzed by flow cytometry. Numbers in the plots indicate the mean fluorescence intensity (MFI) of MHCII on DCs. The MFI values of MHCII in **g,** LNs or **i,** lungs are also summarized in the graph. The data were analyzed by **a-g,** one-way ANOVA with *post hoc* Tukey's test or **i,** two-way ANOVA with *post hoc* Tukey's test. Plots show the mean ± SEM. The data are representative of at least two independent experiments. Source data are provided as a Source Data file.

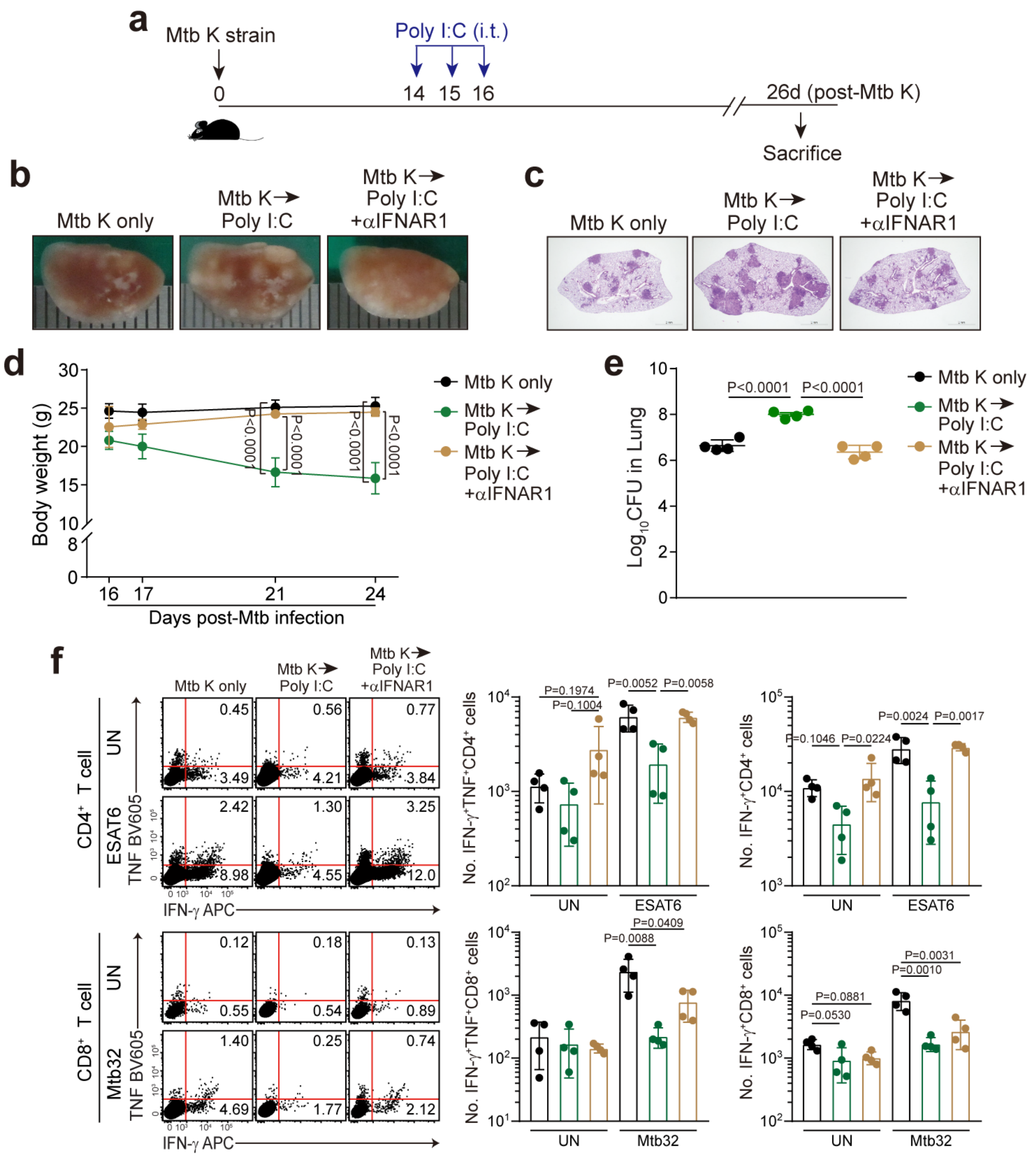
a
Mtb K strain
Poly I:C (i.t.)
0
14 15 16
26d (post-Mtb K)
Sacrifice
b
Mtb K only
Mtb K→ Poly I:C
Mtb K→ Poly I:C +αIFNAR1
c
Mtb K only
Mtb K→ Poly I:C
Mtb K→ Poly I:C +αIFNAR1
d
Body weight (g)
Days post-Mtb infection
P<0.0001
Mtb K only
Mtb K→ Poly I:C
Mtb K→ Poly I:C +αIFNAR1
e
Log10CFU in Lung
P<0.0001
Mtb K only
Mtb K→ Poly I:C
Mtb K→ Poly I:C +αIFNAR1
f
Mtb K only
Mtb K→ Poly I:C
Mtb K→ Poly I:C +αIFNAR1
CD4+ T cell
UN
ESAT6
TNF BV605
IFN-γ APC
CD8+ T cell
Mtb32
No. IFN-γ+TNF+CD4+ cells
No. IFN-γ+CD4+ cells
No. IFN-γ+TNF+CD8+ cells
No. IFN-γ+CD8+ cells
P=0.1974
P=0.1004
P=0.0052
P=0.0058
P=0.1046
P=0.0224
P=0.0024
P=0.0017
P=0.0409
P=0.0088
P=0.0881
P=0.0530
P=0.0031
P=0.0010

Supplementary Figure 7. Exacerbated pulmonary pathology of the lungs of mice injected with poly I:C. a, C57BL/6 mice were infected with Mtb. Some mice from each group were intratracheally injected with 200 μg of poly I:C three times. The mice were sacrificed at 26 days post Mtb infection. **b,** Gross pathology or **c,** H&E staining of the lungs in each group. Scale Bars, 2 mm. **d,** The weight of mice in each group (n=4). **e,** Bacterial loads in each group (n=4). **f,** At 21 days post Mtb infection, isolated lung lymphocytes were restimulated *ex vivo* with ESAT6 peptide pool for $CD4^+$ T cells or Mtb32 peptide pool for $CD8^+$ T cells (n=4). The number of IFN-γ- and TNF-producing $CD4^+$ T cells or $CD8^+$ T cells were analyzed by flow cytometry. **a-f,** The data are representative of a single experiment . The data were analyzed by **d,** two-way ANOVA with *post hoc* Tukey's test or **e-f,** one-way ANOVA with *post hoc* Tukey's test. Source data are provided as a Source Data file.

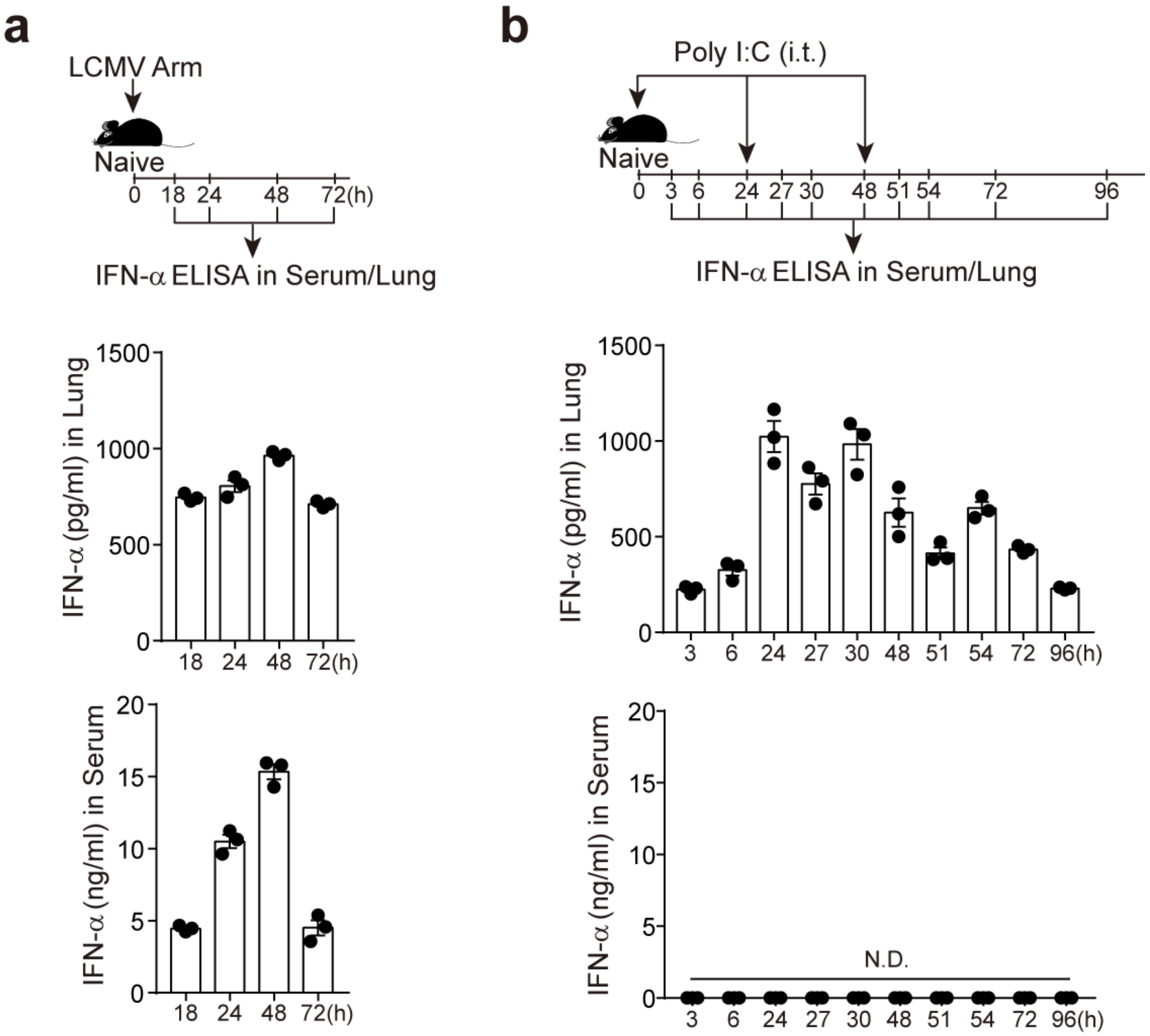
a
LCMV Arm
Naive
0 18 24 48 72(h)
IFN-α ELISA in Serum/Lung
IFN-α (pg/ml) in Lung
1500
1000
500
0
18 24 48 72(h)
IFN-α (ng/ml) in Serum
20
15
10
5
0
18 24 48 72(h)
b
Poly I:C (i.t.)
Naive
0 3 6 24 27 30 48 51 54 72 96
IFN-α ELISA in Serum/Lung
IFN-α (pg/ml) in Lung
1500
1000
500
0
3 6 24 27 30 48 51 54 72 96(h)
IFN-α (ng/ml) in Serum
20
15
10
5
0
N.D.
3 6 24 27 30 48 51 54 72 96(h)

Supplementary Figure 8. The type I IFN expression level by LCMV Arm infection or poly I:C injection

Naïve C57BL/6 mice were **a,** infected with LCMV Arm or **b,** injected with poly I:C. Sera and lung lysates were collected at the indicated time points, and IFN-α levels were analyzed by ELISA. Plots show the mean ± SEM. The data are representative of a single experiment (n=3 mice/group). Source data are provided as a Source Data file.

a

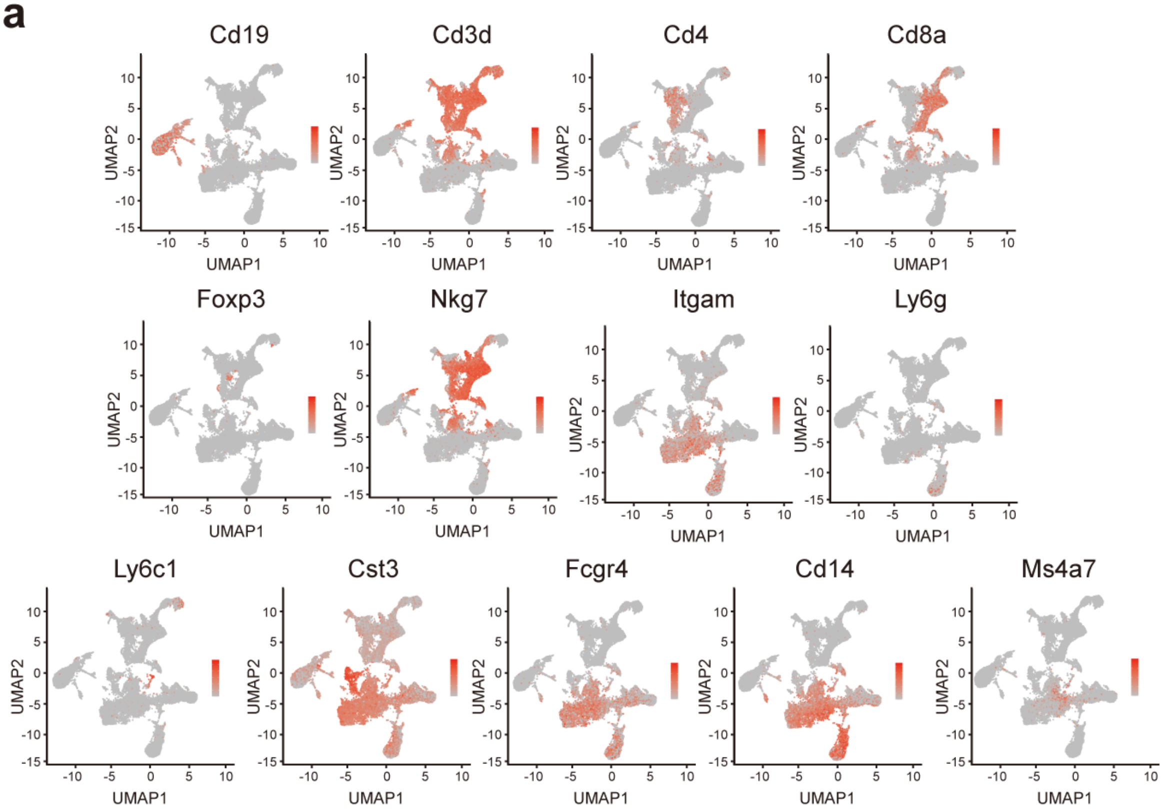

Cd19
Cd3d
Cd4
Cd8a
Foxp3
Nkg7
Itgam
Ly6g
Ly6c1
Cst3
Fcgr4
Cd14
Ms4a7
UMAP1
UMAP2

Supplementary Figure 9. The markers indicating each cell population. a, UMAP plots showing the expression of each marker, characterizing major immune cell populations.

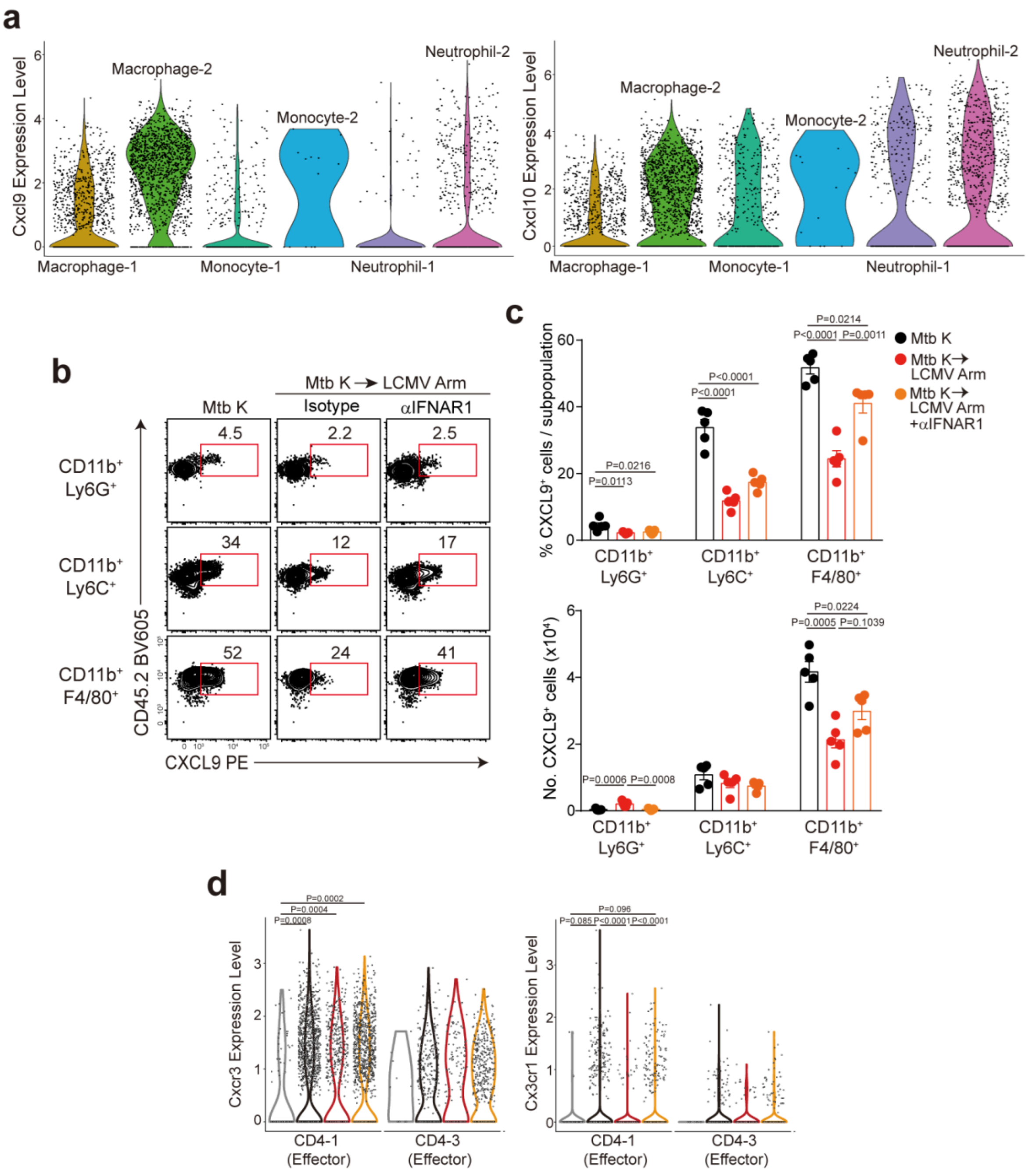
a
Cxcl9 Expression Level
Macrophage-2
Monocyte-2
Neutrophil-2
Macrophage-1
Monocyte-1
Neutrophil-1
Cxcl10 Expression Level
b
Mtb K → LCMV Arm
Mtb K
Isotype
αIFNAR1
CD11b+ Ly6G+
CD11b+ Ly6C+
CD11b+ F4/80+
4.5
2.2
2.5
34
12
17
52
24
41
CD45.2 BV605
CXCL9 PE
c
% CXCL9+ cells / subpopulation
No. CXCL9+ cells (x10^4)
Mtb K
Mtb K→ LCMV Arm
Mtb K→ LCMV Arm +αIFNAR1
P=0.0216
P=0.0113
P<0.0001
P<0.0001
P=0.0214
P<0.0001
P=0.0011
P=0.0224
P=0.0005
P=0.1039
P=0.0006
P=0.0008
d
Cxcr3 Expression Level
Cx3cr1 Expression Level
P=0.0002
P=0.0004
P=0.0008
P=0.096
P=0.085
P<0.0001
P<0.0001
CD4-1 (Effector)
CD4-3 (Effector)

Supplementary Figure 10. The expression of CXCL9 and CXCL10 in myeloid clusters and their receptors in CD4$^+$ T cell effector clusters. a, Violin plots showing the expression of the indicated genes among the indicated myeloid clusters in the Mtb K-only infected group. **(b-c)** Lung myeloid cells were analyzed at 7 days post LCMV Arm infection. **b,** Representative flow cytometry plots of CXCL9$^+$ cells. Numbers in the plots indicate the percentage of CXCL9$^+$ cells in each cell population. **c,** The frequency and number of CXCL9$^+$ cells among the indicated cell populations n=5). **d,** Violin plots showing the expression of the indicated marker genes from the indicated T cell clusters. **b-c,** The data are representative of a single experiment. The data were analyzed by **c,** one-way ANOVA with *post hoc* Tukey's test or **d,** two way with Wilcoxon rank sum test. Plots show the mean ± SEM. Source data are provided as a Source Data file.

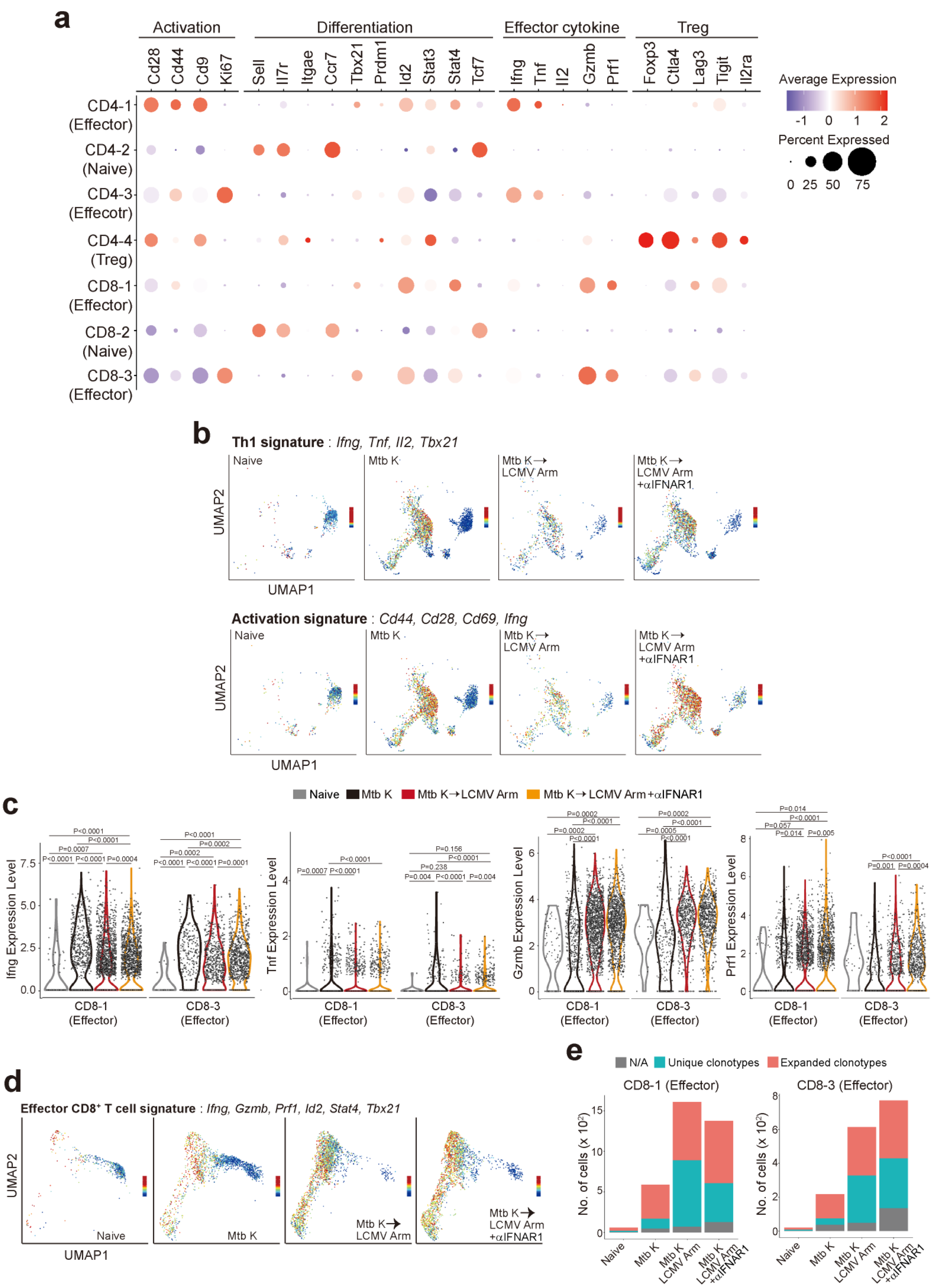
a
Activation
Differentiation
Effector cytokine
Treg
Cd28
Cd44
Cd9
Ki67
Sell
Il7r
Itgae
Ccr7
Tbx21
Prdm1
Id2
Stat3
Stat4
Tcf7
Ifng
Tnf
Il2
Gzmb
Prf1
Foxp3
Ctla4
Lag3
Tigit
Il2ra
CD4-1 (Effector)
CD4-2 (Naive)
CD4-3 (Effecotr)
CD4-4 (Treg)
CD8-1 (Effector)
CD8-2 (Naive)
CD8-3 (Effector)
Average Expression
-1 0 1 2
Percent Expressed
0 25 50 75
b
Th1 signature : Ifng, Tnf, Il2, Tbx21
Naive
Mtb K
Mtb K→ LCMV Arm
Mtb K→ LCMV Arm +αIFNAR1
UMAP2
UMAP1
Activation signature : Cd44, Cd28, Cd69, Ifng
c
Naive
Mtb K
Mtb K→LCMV Arm
Mtb K→ LCMV Arm+αIFNAR1
Ifng Expression Level
Tnf Expression Level
Gzmb Expression Level
Prf1 Expression Level
CD8-1 (Effector)
CD8-3 (Effector)
d
Effector CD8+ T cell signature : Ifng, Gzmb, Prf1, Id2, Stat4, Tbx21
e
N/A
Unique clonotypes
Expanded clonotypes
CD8-1 (Effector)
CD8-3 (Effector)
No. of cells (x 10²)

Supplementary Figure 11. Alteration of T cell signatures by coinfection. a, Dot plots of selected genes. The size of the dots represents the percentage of cells expressing the indicated gene, and color represents the average expression of each indicated gene. **b,** UMAP plots of mean gene expression for Th1 and activation signatures. **c,** Violin plots showing the expression of the indicated marker genes from the indicated T cell clusters. **d,** UMAP plots of mean gene expression for the effector $CD8^+$ T cell signature. **e,** The number of unique or expanded clonotype cells in the effector $CD8^+$ T cell clusters is summarized in the bar graph. N/A indicates "not available" populations, which means that the gray bar represents the nonunique or expanded clonotypes. The $CD45^+$ immune cells were pooled from mice (n=3) in each group. The data were analyzed by two way with Wilcoxon rank sum test.

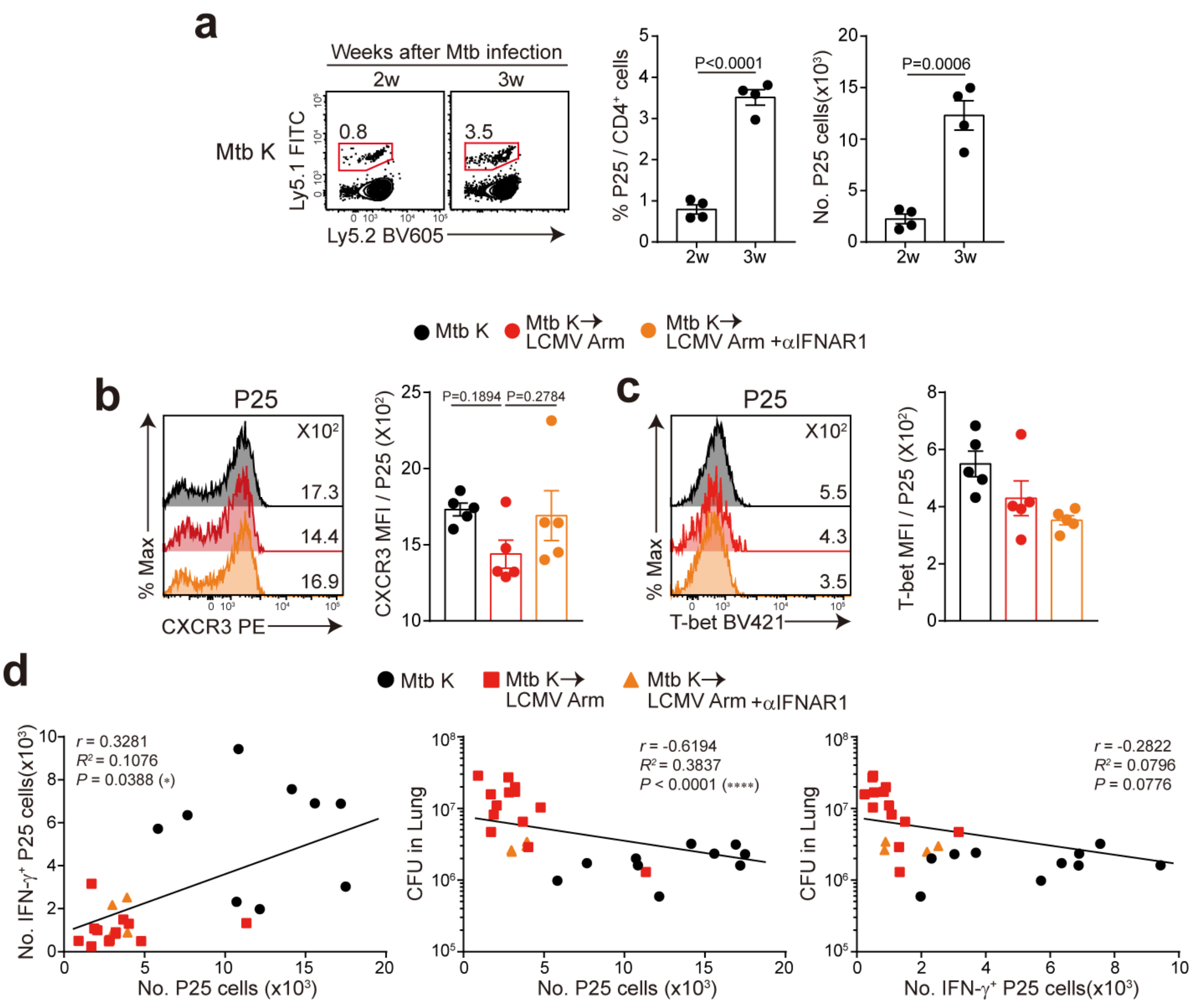
a
Weeks after Mtb infection
2w
3w
Mtb K
Ly5.1 FITC
Ly5.2 BV605
0.8
3.5
% P25 / CD4+ cells
P<0.0001
No. P25 cells(x10³)
P=0.0006
Mtb K
Mtb K→ LCMV Arm
Mtb K→ LCMV Arm +αIFNAR1
b
P25
X10²
17.3
14.4
16.9
% Max
CXCR3 PE
CXCR3 MFI / P25 (X10²)
P=0.1894
P=0.2784
c
P25
X10²
5.5
4.3
3.5
% Max
T-bet BV421
T-bet MFI / P25 (X10²)
d
Mtb K
Mtb K→ LCMV Arm
Mtb K→ LCMV Arm +αIFNAR1
No. IFN-γ+ P25 cells(x10³)
r = 0.3281
R² = 0.1076
P = 0.0388 (*)
No. P25 cells (x10³)
CFU in Lung
r = -0.6194
R² = 0.3837
P < 0.0001 (****)
No. P25 cells (x10³)
CFU in Lung
r = -0.2822
R² = 0.0796
P = 0.0776
No. IFN-γ+ P25 cells(x10³)

Supplementary Figure 12. Analysis of kinetics, markers, and correlation of P25 cells upon blockade of type I IFN signaling. Naïve $CD4^+$ T cells were isolated from the spleens of P25 mice ($Ly5.1^+$) and adoptively transferred via i.v. injection to naïve mice ($Ly5.2^+$). At 1 day after transfer, the mice were infected with Mtb. **a,** At 2 and 3 weeks after Mtb infection, the frequency and number of P25 cells were analyzed (n=4). **b-c,** Some mice from each group were subsequently infected 14 days later with LCMV Arm. At -1 and 1 day after LCMV Arm infection, anti-IFNAR-1 antibodies were administered i.p. into coinfected mice. The mice were sacrificed at 21 days post Mtb infection. The MFIs of CXCR3 and T-bet in P25 cells were analyzed by flow cytometry (n=5). **d,** The correlation between the number of P25 cells or IFN-γ^+ P25 cells in the lung and bacterial loads was analyzed. The data were analyzed by **a,** two-tailed unpaired Student's *t* test or **b, c,** one-way ANOVA with *post hoc* Tukey's test or **d,** two-way test with Pearson's correlation. Plots show the mean ± SEM. **a-c,** The data are representative of a single experiment or **d,** pooled from two or three independent experiments. Source data are provided as a Source Data file.

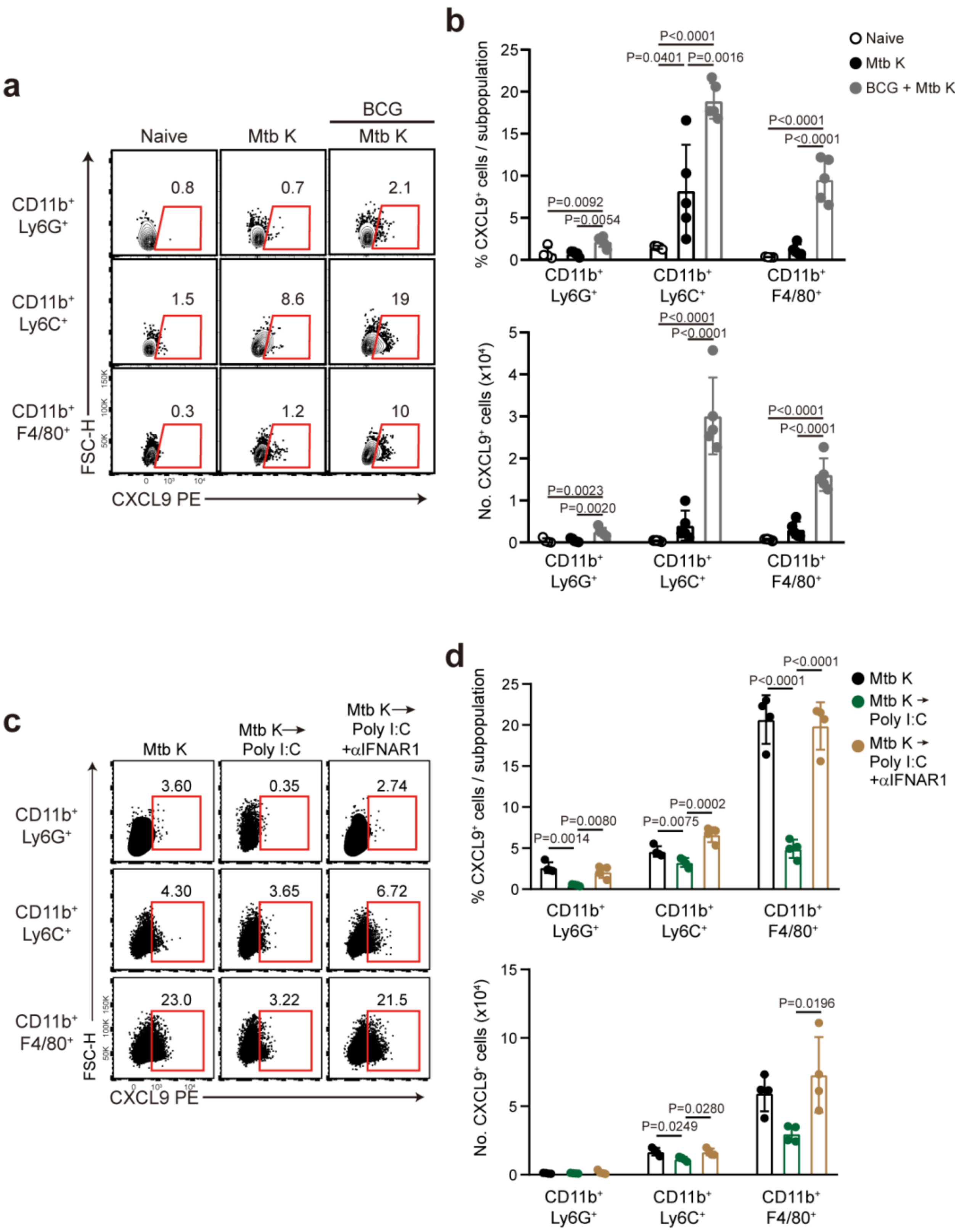
a
Naive
Mtb K
BCG
Mtb K
CD11b+ Ly6G+
CD11b+ Ly6C+
CD11b+ F4/80+
0.8
0.7
2.1
1.5
8.6
19
0.3
1.2
10
FSC-H
CXCL9 PE
b
% CXCL9+ cells / subpopulation
No. CXCL9+ cells (x10⁴)
Naive
Mtb K
BCG + Mtb K
P=0.0092
P=0.0054
P=0.0401
P=0.0016
P<0.0001
P<0.0001
P=0.0023
P=0.0020
c
Mtb K
Mtb K→ Poly I:C
Mtb K→ Poly I:C +αIFNAR1
3.60
0.35
2.74
4.30
3.65
6.72
23.0
3.22
21.5
d
P=0.0014
P=0.0080
P=0.0075
P=0.0002
P=0.0196
P=0.0249
P=0.0280

Supplementary Figure 13. CXCL9 expression induction by BCG vaccination or poly I:C injection. a-b, C57BL/6 mice were vaccinated 3 months before challenge with Mtb. At 14 days post Mtb infection (before LCMV Arm infection), lung myeloid cells were analyzed. **a,** Representative flow cytometry plots of $CXCL9^+$ cells. Numbers in the plots indicate the percentage of $CXCL9^+$ cells in each cell population. **b,** The frequency and number of $CXCL9^+$ cells among the indicated cell populations. **c-d,** C57BL/6 mice were infected with Mtb. Some mice from each group were intratracheally injected with 200 μg of poly I:C three times. The mice were sacrificed at 21 days post Mtb infection and lung myeloid cells were analyzed. **c,** Representative flow cytometry plots of $CXCL9^+$ cells. Numbers in the plots indicate the percentage of $CXCL9^+$ cells in each cell population. **d,** The frequency and number of $CXCL9^+$ cells among the indicated cell populations. The data were analyzed by one-way ANOVA with *post hoc* Tukey's test. Plots show the mean ± SEM. The data are representative of a single experiment (n=4 mice/group). Source data are provided as a Source Data file.

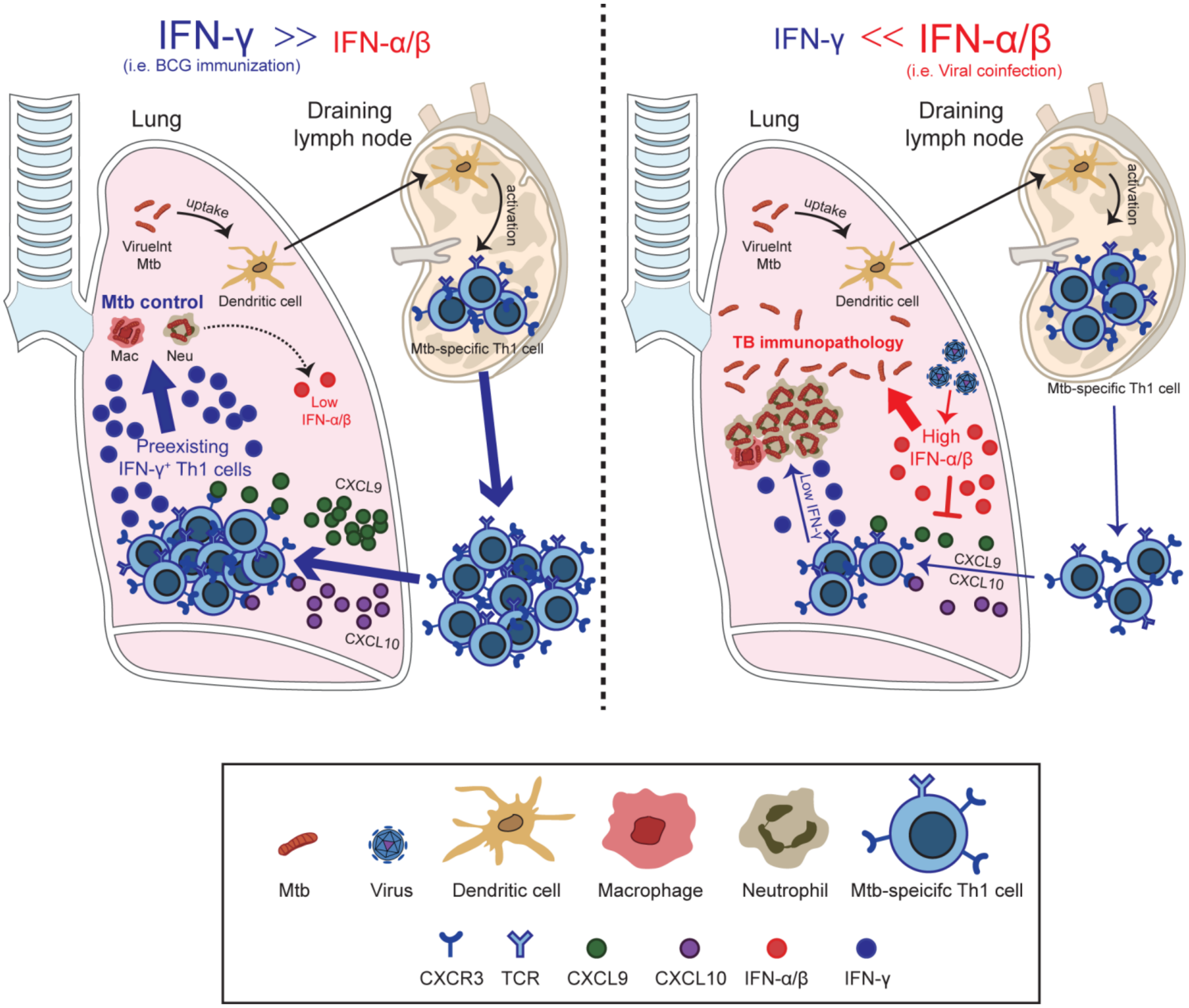
IFN-γ >> IFN-α/β
(i.e. BCG immunization)
Lung
Draining lymph node
uptake
VirueInt Mtb
Dendritic cell
activation
Mtb control
Mac
Neu
Mtb-specific Th1 cell
Low IFN-α/β
Preexisting IFN-γ⁺ Th1 cells
CXCL9
CXCL10
IFN-γ << IFN-α/β
(i.e. Viral coinfection)
Lung
Draining lymph node
uptake
VirueInt Mtb
Dendritic cell
activation
TB immunopathology
Mtb-specific Th1 cell
High IFN-α/β
Low IFN-γ
CXCL9
CXCL10
Mtb
Virus
Dendritic cell
Macrophage
Neutrophil
Mtb-speicifc Th1 cell
CXCR3
TCR
CXCL9
CXCL10
IFN-α/β
IFN-γ

Supplementary Figure 14. Graphical abstract of our experiments. (Left) When Mtb-specific Th1 responses preexisted under some conditions, such as BCG immunization, Mtb-specific Th1 cells were properly activated and proliferated in draining lymph nodes and then migrated to pulmonary lesions to protect the host from Mtb. **(Right)** On the other hand, when mice were exposed to high levels of type I IFN production, such as during LCMV infection *in vivo*, migration of Mtb-specific Th1 cells was hindered by downregulation of CXCL9/10 expression in a type I IFN-dependent manner. The reduced number of Mtb-specific Th1 cells in pulmonary lesions triggered Mtb dissemination and pathological exacerbation due to low levels of IFN-γ in Mtb-specific T cells.
