# Supplementary material for: Viral coinfection promotes tuberculosis immunopathogenesis by type I IFN signaling-dependent impediment of Th1 cell pulmonary influx
Source: Nat Commun. 2022 Jun 7;13:3155. doi: 10.1038/s41467-022-30914-3 (PMC9174268; doi:10.1038/s41467-022-30914-3)
Supplement: Supplementary file 3 — Reporting Summary [file 41467_2022_30914_MOESM3_ESM.pdf]

nature portfolio

Corresponding author(s): Sung Jae Shin

Last updated by author(s): 2022.04.22

Reporting Summary

Nature Portfolio wishes to improve the reproducibility of the work that we publish. This form provides structure for consistency and transparency in reporting. For further information on Nature Portfolio policies, see our Editorial Policies and the Editorial Policy Checklist.

Statistics

For all statistical analyses, confirm that the following items are present in the figure legend, table legend, main text, or Methods section.

n/a	Confirmed	
☐	☒	The exact sample size (*n*) for each experimental group/condition, given as a discrete number and unit of measurement
☐	☒	A statement on whether measurements were taken from distinct samples or whether the same sample was measured repeatedly
☐	☒	The statistical test(s) used AND whether they are one- or two-sided<br>*Only common tests should be described solely by name; describe more complex techniques in the Methods section.*
☒	☐	A description of all covariates tested
☐	☒	A description of any assumptions or corrections, such as tests of normality and adjustment for multiple comparisons
☐	☒	A full description of the statistical parameters including central tendency (e.g. means) or other basic estimates (e.g. regression coefficient) AND variation (e.g. standard deviation) or associated estimates of uncertainty (e.g. confidence intervals)
☐	☒	For null hypothesis testing, the test statistic (e.g. *F*, *t*, *r*) with confidence intervals, effect sizes, degrees of freedom and *P* value noted<br>*Give P values as exact values whenever suitable.*
☒	☐	For Bayesian analysis, information on the choice of priors and Markov chain Monte Carlo settings
☒	☐	For hierarchical and complex designs, identification of the appropriate level for tests and full reporting of outcomes
☐	☒	Estimates of effect sizes (e.g. Cohen's *d*, Pearson's *r*), indicating how they were calculated

Our web collection on statistics for biologists contains articles on many of the points above.

Software and code

Policy information about availability of computer code

Data collection	H&E image : Olympus BX43 microscope (Olympus Optical Co.)<br>qRT-PCR : CFX96 real-time PCR detection system (Bio-Rad)<br>Multiplex IHC : Vectra Polaris Automated Quantitative Pathology Imaging system (Akoya Bioscience)<br>FACS analysis : FACS Canto II (BD bioscience) & CytoFLEX LX (Beckman Coulter)<br>scRNA & scTCR seq. : HiseqX platform (Illumina)
Data analysis	For inflammation area calculation, data were calculated by ImageJ (v1.53, NIH), ToukTek Toupr viewer program (v4.11, Toup View Co., Zhejiang , China)<br>For multiplex IHC images, data were analyzed by inform 2.4 and TIBCO Spotfire (Akoya Bioscience)<br>For FACS data, data were analyzed by FlowJo software (v10.5.3, TreeStar)<br>For statistical analysis, data were analyzed by Graphpad Prism 7.00 (v3.6.1, GraphPad)<br><br>For scRNA-seq & scTCR-seq data analysis, we used standard published algorithms and these were described below;<br>(Also, these description were written in the manuscript word file in page 35)<br><br>1. Data were analyze by Seurat R package (R Foundation for statistical, v3.6.1)<br>Reference 93 in the manuscript. Butler, A., Hoffman, P., Smibert, P., Papalexi, E. & Satija, R. Integrating single-cell transcriptomic data across different conditions, technologies, and species. Nat. Biotechnol. 36, 411–420 (2018).<br>Reference 100 in the manuscript. Stuart, T. et al. Comprehensive integration of single-cell data. Cell 177, 1888–1902.e1821 (2019).<br>2. Data were analyzed by Seurat (v4.0.0)<br>Reference 101 in the manuscript. Blondel, V.D., Guillaume, J.-L., Lambiotte, R. & Lefebvre, E. Fast unfolding of communities in large networks. J. Stat. Mech. Theory Exp. 2008, P10008 (2008).

3. Data were analyzed by scMC (v1.0.0)
Reference 94 in the manuscript. Zhang, L. & Nie, Q. scMC learns biological variation through the alignment of multiple single-cell genomics datasets. Genome Biol. 22, 10 (2021).

For manuscripts utilizing custom algorithms or software that are central to the research but not yet described in published literature, software must be made available to editors and reviewers. We strongly encourage code deposition in a community repository (e.g. GitHub). See the Nature Portfolio guidelines for submitting code & software for further information.

Data

Policy information about availability of data

All manuscripts must include a data availability statement. This statement should provide the following information, where applicable:

- Accession codes, unique identifiers, or web links for publicly available datasets
- A description of any restrictions on data availability
- For clinical datasets or third party data, please ensure that the statement adheres to our policy

The scRNA-seq and scTCR seq data generated in this study have been deposited in the Gene Expression Omnibus database under accession code GSE167650. The deposited data are available at "https://www.ncbi.nlm.nih.gov/geo/query/acc.cgi?acc=GSE167650". Other source data are provided with this paper in the Source Data file.

Field-specific reporting

Please select the one below that is the best fit for your research. If you are not sure, read the appropriate sections before making your selection.

[x] Life sciences [ ] Behavioural & social sciences [ ] Ecological, evolutionary & environmental sciences

For a reference copy of the document with all sections, see nature.com/documents/nr-reporting-summary-flat.pdf

Life sciences study design

All studies must disclose on these points even when the disclosure is negative.

Sample size	We used sample sizes containing 3 or more biological replicates which can provide adequate statistical power in biological analysis. In addition, for both in vitro and in vivo experiments, the group sizes were also selected empirically based upon our prior knowledge of responses of these cells and animals. We have described the exact sample sizes and their statistic significance for each experiment in our manuscript.
Data exclusions	For in vivo animal experiment, no data exclusions were needed. For scRNA & scTCR seq, cells that did not pass standard single cell QC (not CD45+ cells, doublets, dead cells) were excluded.
Replication	Almost experiments were conducted at least 2 times. And these replicated experiments were reliably reproduced (described in each figure legends for further details).
Randomization	Samples and mice were randomly assigned to the different experimental groups
Blinding	No blinded experiments were conducted in this study, because this study was an observational study.

Reporting for specific materials, systems and methods

We require information from authors about some types of materials, experimental systems and methods used in many studies. Here, indicate whether each material, system or method listed is relevant to your study. If you are not sure if a list item applies to your research, read the appropriate section before selecting a response.

Materials & experimental systems

n/a	Involved in the study	
[ ]	[x]	Antibodies
[ ]	[x]	Eukaryotic cell lines
[x]	[ ]	Palaeontology and archaeology
[ ]	[x]	Animals and other organisms
[x]	[ ]	Human research participants
[x]	[ ]	Clinical data
[x]	[ ]	Dual use research of concern

Methods

n/a	Involved in the study	
[x]	[ ]	ChIP-seq
[ ]	[x]	Flow cytometry
[x]	[ ]	MRI-based neuroimaging

Antibodies

Antibodies used	Dye Antibody (Clone), Manufacturer, Catalog number

The following antibodies were against mouse proteins:
APC Anti-Mouse CD90.2 (clone 53-2.1), BD Bioscience, Cat#553007
PE-Cy7 Anti-Mouse CD11b (clone M1/70), BD Bioscience, Cat#552850
FITC Anti-Mouse CD11c (clone HL3), BD Bioscience, Cat#561045
BV605 Anti-Mouse Ly-6C (clone AL-21), BD Bioscience, Cat#563011
PerCP-Cy5.5 Anti-Mouse Ly-6G (clone 1A8), BD Bioscience, Cat#560602
APC Anti-Mouse NK1.1 (clone PK136), BD Bioscience, Cat#561117
Alexa Fluor 488 Anti-Mouse Ki-67 (clone B56), BD Bioscience, Cat#558616
APC Anti-Mouse IFN-γ (clone XMG1.2), BD Bioscience, Cat#554413
APC Anti-Mouse NKG2D (clone CX5), BD Bioscience, Cat#562347

PerCP-Cy5.5 Anti-Mouse CD8a (clone 53-6.7), Thermo Fisher, Cat#45-0081-80
APC Anti-Mouse CD19 (clone eBio1D3), Thermo Fisher, Cat#17-0193-80
PE-Cy7 Anti-Mouse CD44 (clone IM7), Thermo Fisher, Cat#25-0441-82
eFluor 450 Anti-Mouse MHC Class II (clone M5/114.15.2), Thermo Fisher, Cat#48-5321-82
APC Anti-Mouse Foxp3 (clone FJK-16s), Thermo Fisher, Cat#17-5773-82
PE Anti-Mouse CXCR3 (clone CXCR3-173), Thermo Fisher, Cat#12-1831-82

BV421 Anti-Mouse CD4 (clone RM4-5), Biolegend, Cat#100543
FITC Anti-Mouse CD45.1 (clone A20), Biolegend, Cat#110705
BV605 Anti-Mouse CD45.2 (clone 104), Biolegend, Cat#109841
PE Anti-Mouse CD25 (clone PC61), Biolegend, Cat#102007
BV605 Anti-Mouse TNF-α (MP6-XT22), Biolegend, Cat#506329
PE Anti-Mouse CXCL9 (MIG-2F5.5), Biolegend, Cat#515604
BV421 Anti-Mouse T-bet(4B10), Biolegend, Cat#644816

Anti-Mouse IFNAR-1 (clone MAR1-5A3), Bio X cell, Cat#BE0241
Anti-Mouse CD8 (clone 53-6.7), Bio X cell, Cat#BE0004
Anti-Mouse Ly6G/Ly6C (clone RB6-8C5), Bio X cell, Cat#BE0075
Anti-Mouse IL-10R (clone 1B1.3A), Bio X cell, Cat#BE0050
Anti-Mouse NKG2D (clone HMG2D), Bio X cell, Cat#BE0111
Anti-Mouse CD4 (clone GK1.5), Bio X cell, Cat#BE0003
Anti-Mouse IL-1R (clone JAMA-147), Bio X cell, Cat#BE0256
Anti-Mouse IL-6R (clone 15A7), Bio X cell, Cat#BE0047
Anti-Mouse TNFα (clone XT3.11), Bio X cell, Cat#BE0058
Anti-Mouse Ly6G (clone 1A8), Bio X cell, Cat#BE0075
Mouse IgG1 isotype control (clone MOPC-21), Bio X cell, Cat#BE0083
Rat IgG2a isotype control (clone 2A3), Bio X cell, Cat#BE0089
Rat IgG2b isotype control (clone LTF-2), Bio X cell, Cat#BE0090
Rat IgG1 isotype control (clone HRPN), Bio X cell, Cat#BE0088
Polyclonal Armenian hamster IgG ,Bio X cell, Cat#BE0091
Purified NA/LE Hamster Anti-Mouse CD3e (clone145-2C11), BD Bioscience, Cat#553057
Purified NA/LE Hamster Anti-Mouse CD28 (clone 37.51), BD Bioscience, Cat#553294
Anti-Mouse CD45.2 (clone 104-2), Miltenyi Biotec, Cat#130-102-458
Anti-Mouse CD31 (polyclonal), Abcam, Cat#ab124432
Anti-Mouse CXCL9 (polyclonal), Abcam, Cat#ab202961
Anti-Mouse CD11b (clone EPR1344), Abcam, Cat#ab133357
Anti-Mouse PDPN (polyclonal), Abcam, Cat#ab109059

Validation

Dye Antibody (Clone), Manufacturer, Catalog number
All antibodies are from commercial sources and the validation data are available on website of the manufactures. The application of all antibodies were followed by the instructions of the website. The following antibodies were against mouse proteins:
APC Anti-Mouse CD90.2 (clone 53-2.1), BD Bioscience, Cat#553007:https://www.bdbiosciences.com/en-us/products/reagents/flow-cytometry-reagents/research-reagents/single-color-antibodies-ruo/apc-rat-anti-mouse-cd90-2.553007
PE-Cy7 Anti-Mouse CD11b (clone M1/70), BD Bioscience, Cat#552850:https://www.bdbiosciences.com/en-us/products/reagents/flow-cytometry-reagents/research-reagents/single-color-antibodies-ruo/pe-cy-7-rat-anti-cd11b.552850
FITC Anti-Mouse CD11c (clone HL3), BD Bioscience, Cat#561045:https://www.bdbiosciences.com/en-us/products/reagents/flow-cytometry-reagents/research-reagents/single-color-antibodies-ruo/fitc-hamster-anti-mouse-cd11c.561045
BV605 Anti-Mouse Ly-6C (clone AL-21), BD Bioscience, Cat#563011:https://www.bdbiosciences.com/en-us/products/reagents/flow-cytometry-reagents/research-reagents/single-color-antibodies-ruo/bv605-rat-anti-mouse-ly-6c.563011
PerCP-Cy5.5 Anti-Mouse Ly-6G (clone 1A8), BD Bioscience, Cat#560602:https://www.bdbiosciences.com/en-us/products/reagents/flow-cytometry-reagents/research-reagents/single-color-antibodies-ruo/percp-cy-5-5-rat-anti-mouse-ly-6g.560602
APC Anti-Mouse NK1.1 (clone PK136), BD Bioscience, Cat#561117:https://www.bdbiosciences.com/en-us/products/reagents/flow-cytometry-reagents/research-reagents/single-color-antibodies-ruo/apc-mouse-anti-mouse-nk-1-1.561117
Alexa Fluor 488 Anti-Mouse Ki-67 (clone B56), BD Bioscience, Cat#558616:https://www.bdbiosciences.com/en-us/products/reagents/microscopy-imaging-reagents/immunofluorescence-reagents/alexa-fluor-488-mouse-anti-ki-67.558616
APC Anti-Mouse IFN-γ (clone XMG1.2), BD Bioscience, Cat#554413:https://www.bdbiosciences.com/en-us/products/reagents/flow-

cytometry-reagents/research-reagents/single-color-antibodies-ruo/apc-rat-anti-mouse-ifn.554413
APC Anti-Mouse NKG2D (clone CX5), BD Bioscience, Cat#562347:https://www.bdbiosciences.com/en-us/products/reagents/flow-cytometry-reagents/research-reagents/single-color-antibodies-ruo/apc-rat-anti-mouse-cd314.562347

PerCP-Cy5.5 Anti-Mouse CD8a (clone 53-6.7), Thermo Fisher, Cat#45-0081-80:https://www.thermofisher.com/antibody/product/CD8a-Antibody-clone-53-6-7-Monoclonal/45-0081-80
APC Anti-Mouse CD19 (clone eBio1D3), Thermo Fisher, Cat#17-0193-80:https://www.thermofisher.com/antibody/product/CD19-Antibody-clone-eBio1D3-1D3-Monoclonal/17-0193-80
PE-Cy7 Anti-Mouse CD44 (clone IM7), Thermo Fisher, Cat#25-0441-82:https://www.thermofisher.com/antibody/product/CD44-Antibody-clone-IM7-Monoclonal/25-0441-82
eFluor 450 Anti-Mouse MHC Class II (clone M5/114.15.2), Thermo Fisher, Cat#48-5321-82:https://www.thermofisher.com/antibody/product/MHC-Class-II-I-A-I-E-Antibody-clone-M5-114-15-2-Monoclonal/48-5321-82
APC Anti-Mouse Foxp3 (clone FJK-16s), Thermo Fisher, Cat#17-5773-82:https://www.thermofisher.com/antibody/product/FOXP3-Antibody-clone-FJK-16s-Monoclonal/17-5773-82
PE Anti-Mouse CXCR3 (clone CXCR3-173), Thermo Fisher, Cat#12-1831-82:https://www.thermofisher.com/antibody/product/CD183-CXCR3-Antibody-clone-CXCR3-173-Monoclonal/12-1831-82

BV421 Anti-Mouse CD4 (clone RM4-5), Biolegend, Cat#100543:https://www.biolegend.com/en-us/products/brilliant-violet-421-anti-mouse-cd4-antibody-7349
FITC Anti-Mouse CD45.1 (clone A20), Biolegend, Cat#110705:https://www.biolegend.com/en-us/products/fitc-anti-mouse-cd45-1-antibody-198
BV605 Anti-Mouse CD45.2 (clone 104), Biolegend, Cat#109841:https://www.biolegend.com/en-us/products/brilliant-violet-605-anti-mouse-cd45-2-antibody-9695
PE Anti-Mouse CD25 (clone PC61), Biolegend, Cat#102007:https://www.biolegend.com/en-us/products/pe-anti-mouse-cd25-antibody-424
BV605 Anti-Mouse TNF-α (MP6-XT22), Biolegend, Cat#506329:https://www.biolegend.com/en-us/products/brilliant-violet-605-anti-mouse-tnf-alpha-antibody-7682
PE Anti-Mouse CXCL9 (MIG-2F5.5), Biolegend, Cat#515604:https://www.biolegend.com/en-us/products/pe-anti-mouse-cxcl9-mig-antibody-6147
BV421 Anti-Mouse T-bet(4B10), Biolegend, Cat#644816:https://www.biolegend.com/en-us/products/brilliant-violet-421-anti-t-bet-antibody-7281

Anti-Mouse IFNAR-1 (clone MAR1-5A3), Bio X cell, Cat#BE0241:https://bxcell.com/product/anti-m-ifnar-1/
Anti-Mouse CD8 (clone 53-6.7), Bio X cell, Cat#BE0004:https://bxcell.com/product/m-cd8a/
Anti-Mouse Ly6G/Ly6C (clone RB6-8C5), Bio X cell, Cat#BE0075:https://bxcell.com/product/m-ly-6g-2/
Anti-Mouse IL-10R (clone 1B1.3A), Bio X cell, Cat#BE0050:https://bxcell.com/product/m-il-10r/
Anti-Mouse NKG2D (clone HMG2D), Bio X cell, Cat#BE0111:https://bxcell.com/product/m-nkg2d/
Anti-Mouse CD4 (clone GK1.5), Bio X cell, Cat#BE0003:https://bxcell.com/product/m-cd4/
Anti-Mouse IL-1R (clone JAMA-147), Bio X cell, Cat#BE0256:https://bxcell.com/product/anti-m-il-1-r/
Anti-Mouse IL-6R (clone 15A7), Bio X cell, Cat#BE0047:https://bxcell.com/product/m-il-6r/
Anti-Mouse TNFα (clone XT3.11), Bio X cell, Cat#BE0058:https://bxcell.com/product/m-tnf-alpha/
Anti-Mouse Ly6G (clone 1A8), Bio X cell, Cat#BE0075:https://bxcell.com/product/invivomab-anti-m-ly-6g/
Mouse IgG1 isotype control (clone MOPC-21), Bio X cell, Cat#BE0083:https://bxcell.com/product/mouse-igg1-isotype-control/
Rat IgG2a isotype control (clone 2A3), Bio X cell, Cat#BE0089:https://bxcell.com/product/rat-igg2a-isotype-control/
Rat IgG2b isotype control (clone LTF-2), Bio X cell, Cat#BE0090:https://bxcell.com/product/rat-igg2b-isotype-control/
Rat IgG1 isotype control (clone HRPN), Bio X cell, Cat#BE0088:https://bxcell.com/product/rat-igg1-isotype-control/
Polyclonal Armenian hamster IgG ,Bio X cell, Cat#BE0091:https://bxcell.com/product/polyclonal-3/
Purified NA/LE Hamster Anti-Mouse CD3e (clone145-2C11), BD Bioscience, Cat#553057:https://www.bdbiosciences.com/en-us/products/reagents/flow-cytometry-reagents/research-reagents/single-color-antibodies-ruo/purified-na-le-hamster-anti-mouse-cd3e.553057
Purified NA/LE Hamster Anti-Mouse CD28 (clone 37.51), BD Bioscience, Cat#553294:https://www.bdbiosciences.com/en-us/products/reagents/flow-cytometry-reagents/research-reagents/single-color-antibodies-ruo/purified-na-le-hamster-anti-mouse-cd28.553294
Anti-Mouse CD45.2 (clone 104-2), Miltenyi Biotec, Cat#130-102-458:https://www.miltenyibiotec.com/US-en/products/cd8a-antibody-anti-mouse-53-6-7.html#gref

Anti-Mouse CD31 (polyclonal), Abcam, Cat#ab124432:https://www.abcam.com/cd31-antibody-ab124432.html
Anti-Mouse CXCL9 (polyclonal), Abcam, Cat#ab202961:https://www.abcam.com/cxcl9-antibody-ab202961.html
Anti-Mouse CD11b (clone EPR1344), Abcam, Cat#ab133357:https://www.abcam.com/cd11b-antibody-epr1344-ab133357.html
Anti-Mouse PDPN (polyclonal), Abcam, Cat#ab109059:https://www.abcam.com/podoplanin--gp36-antibody-ab109059.html

Dilution factor information are described in method section of manuscript.

Eukaryotic cell lines

Policy information about cell lines

Cell line source(s)	Monkey: Vero kidney epithelial cells, ATCC ATCC# CCL-81<br>Mouse: mouse monocyte/macrophage cells, ATCC ATCC# TIB-71

Authentication	None of the cell lines were authenticated in these studies. In all related studies, cell lines with low passage number were used.
Mycoplasma contamination	All cell lines were confirmed mycoplasma negative.
Commonly misidentified lines (See ICLAC register)	No commonly misidentified cell lines were used.

Animals and other organisms

Policy information about studies involving animals; ARRIVE guidelines recommended for reporting animal research

Laboratory animals	Mouse: C57BL/6J (6~8 weeks), The Jackson Laboratory, JAX:000664 (Female)<br>Mouse: Ifnar1-/- (B6.129S2-Ifnar1tm1Agt/Mmjax) (6~8 weeks), obtained from laboratory of Heung Kyu Lee of KAIST, JAX:32045 (Female)<br>Mouse: CD45.1 (6~8 weeks)(CD45.1+: B6.SJL-Ptprca Pep3b/BoyJ), obtained from laboratory of Young-Chul Sung of POSTECH, N/A (Female), JAX:002014<br>Mouse: P25 (C57BL/6-Tg(H2-Kb-Tcra, Tcrb)P25Ktk/J) (6~8 weeks), The Jackson Laboratory, JAX:011005 (Female)
Wild animals	No wild animals were involved in this study
Field-collected samples	No field-collected samples were used in this study
Ethics oversight	Animal maintenance and procedures were performed with approval of the IACUC of Yonsei University College of Medicine (Permit number : 2016-0305). All animal studies were performed in accordance with Koran Food and Drug Administration (KFDA) guidelines.

Note that full information on the approval of the study protocol must also be provided in the manuscript.

Flow Cytometry

Plots

Confirm that:

- [x] The axis labels state the marker and fluorochrome used (e.g. CD4-FITC).
- [x] The axis scales are clearly visible. Include numbers along axes only for bottom left plot of group (a 'group' is an analysis of identical markers).
- [x] All plots are contour plots with outliers or pseudocolor plots.
- [x] A numerical value for number of cells or percentage (with statistics) is provided.

Methodology

Sample preparation	To create a single-cell suspension, each lung was chopped into small pieces and incubated in RPMI supplemented with 1.3 mM EDTA at 37°C in a shaking incubator for 30 min. After incubation, the small fragments were incubated again in complete medium solution containing 0.1% collagenase type II (Worthington Biochemical, Lakewood, NJ, USA) for 1 h. The single-cell suspensions were then filtered through a 40-μm cell nylon mesh strainer, treated with red blood cell (RBC) lysis buffer (Gibco) for 3 min, and washed twice with RPMI containing 2% FBS. Lymphocytes from LNs were mashed through a 70-μm cell strainer (BD Falcon), and RBC lysis was performed using ACK lysis buffer (Gibco Laboratories).
Instrument	Cells were analyzed by FACS Canto II (BD bioscience) & CytoFLEX LX (Beckman Coulter).
Software	Flowjo software (ver.10.5.3) was used for data analysis.
Cell population abundance	For CD11b+Ly6G+ suppression assay, Live CD11b+Ly6G+ cells from infected mice were purified to >90% purity.
Gating strategy	We excluded doublets (FSC-H vs FSC-A) and gated based on physical parameters (FSC-A vs SSC-A).<br>On the singlets, we selected for live cells using LIVE/DEAD fixable dead cell stain kit (Invitrogen).<br>Cell populations were identified based on the expression markers listed below<br>CD8+ T cells: CD4-/CD8+<br>CD4+ T cells: CD4+/CD8-<br>DCs: Thy1.2-NK1.1-CD19- / MHCII+ / CD11c+<br>CD11b+Ly6C+: Thy1.2-NK1.1-CD19- / CD11b+ / Ly6C+<br>CD11b+Ly6G+: Thy1.2-NK1.1-CD19- / CD11b+ / Ly6G+<br>CD4+Foxp3+ T cells (Tregs): CD4+/CD8-/Foxp3+

- [x] Tick this box to confirm that a figure exemplifying the gating strategy is provided in the Supplementary Information.
